# Deep learning-based framework for slide-based histopathological image analysis

**DOI:** 10.1038/s41598-022-23166-0

**Published:** 2022-11-09

**Authors:** Sai Kosaraju, Jeongyeon Park, Hyun Lee, Jung Wook Yang, Mingon Kang

**Affiliations:** 1 Department of Computer Science, University of Nevada, Las Vegas, Las Vegas, NV 89154 USA; 2 Department of Computer Science, Sun Moon University, Asan, 336708 South Korea; 3 Department of Pathology, Gyeongsang National University Hospital, Gyeongsang National University College of Medicine, Jinju, South Korea

**Keywords:** Medical research, Mathematics and computing

## Abstract

Digital pathology coupled with advanced machine learning (e.g., deep learning) has been changing the paradigm of whole-slide histopathological images (WSIs) analysis. Major applications in digital pathology using machine learning include automatic cancer classification, survival analysis, and subtyping from pathological images. While most pathological image analyses are based on patch-wise processing due to the extremely large size of histopathology images, there are several applications that predict a single clinical outcome or perform pathological diagnosis per slide (e.g., cancer classification, survival analysis). However, current slide-based analyses are task-dependent, and a general framework of slide-based analysis in WSI has been seldom investigated. We propose a novel slide-based histopathology analysis framework that creates a WSI representation map, called HipoMap, that can be applied to any slide-based problems, coupled with convolutional neural networks. HipoMap converts a WSI of various shapes and sizes to structured image-type representation. Our proposed HipoMap outperformed existing methods in intensive experiments with various settings and datasets. HipoMap showed the Area Under the Curve (AUC) of 0.96±0.026 (5% improved) in the experiments for lung cancer classification, and c-index of 0.787±0.013 (3.5% improved) and coefficient of determination ($R^2$) of 0.978±0.032 (24% improved) in survival analysis and survival prediction with TCGA lung cancer data respectively, as a general framework of slide-based analysis with a flexible capability. The results showed significant improvement comparing to the current state-of-the-art methods on each task. We further discussed experimental results of HipoMap as pathological viewpoints and verified the performance using publicly available TCGA datasets. A Python package is available at https://pypi.org/project/hipomap, and the package can be easily installed using Python PIP. The open-source codes in Python are available at: https://github.com/datax-lab/HipoMap.

## Introduction

Whole-Slide histopathological Images (WSIs) have been considered a clinical gold standard tool for the diagnosis of complex diseases (i.e., cancers)^1,2^, since diagnosis is mainly determined by morphological patterns in WSIs^3^. Recent advances in artificial intelligence techniques in digital pathology have produced significant performances for diagnosis and clinical outcome prediction. Deep learning-based segmentation and classification models have assisted pathologists on diagnosis of complex diseases^4,5^ and identified subtype-related morphologies in WSI^6^. Deep learning-based survival analysis models have shown potential in estimating survival of patients as well as identifying survival-related morphological patterns from WSI^7,8^.

**Figure 1:**
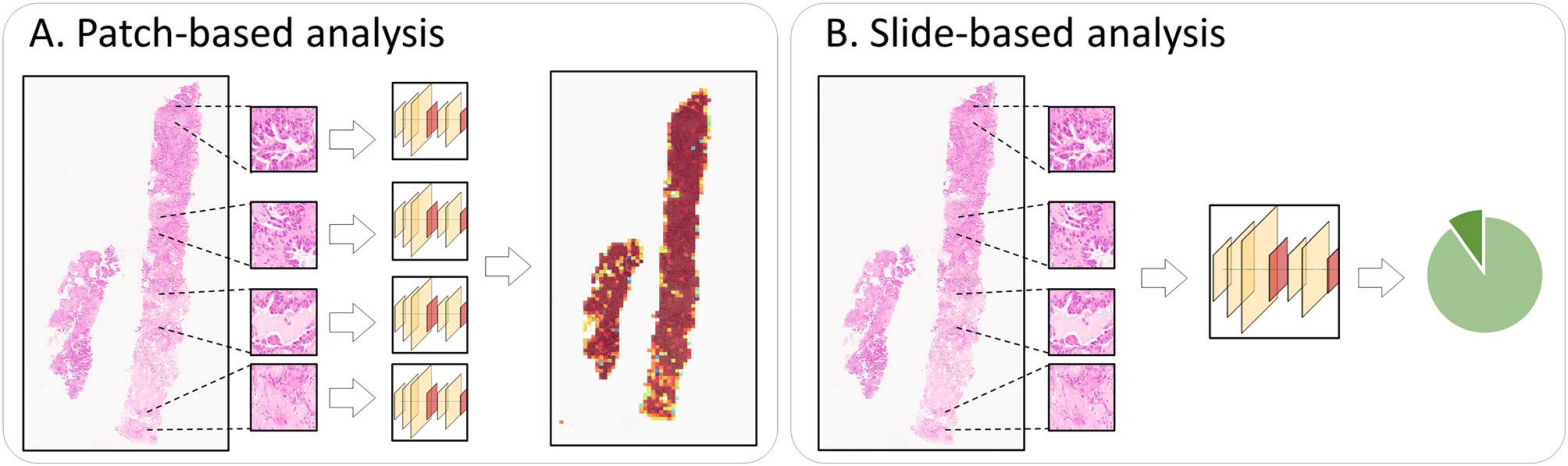
(**A**) Patch-wise and (**B**) slide-based histopathological analyses. Patch-wise analysis produces a probability map, whereas slide-based analysis predict a single clinical outcome or diagnosis.

Most computational approaches on histopathological images are based on patch-wise processing, due to the various shapes of tissues and giga-pixel sizes of WSIs^9,10^ (see Fig. 1A). Patch-wise approaches typically consist of the three steps: (1) a WSI is divided into smaller patches; (2) each of the patches computes a probability score of a diagnosis or a clinical outcome (e.g., a probability that the patch region is cancerous); and (3) the probability scores of the patches are combined into an entire probability map of the WSI^11^. For instance, Convolutional Neural Networks (CNNs) were trained with fixed-size patch images (e.g., $299\times 299$ pixels), and the patch-wise results localized cancerous regions in a WSI^12,13^. Patches in annotated Regions of Interest (ROI) were used to train a CNN-based model to predict risk scores of patches^14,15^. A multi-scale receptive field CNN examined patches of multiple magnification levels simultaneously for cancer recognition^16^. Those patch-wise approaches are widely applicable for identifying regions of interest on a WSI.

On the other hand, slide-based approaches are designed to predict a single clinical outcome or diagnosis for an entire WSI (see Fig. 1B). Typically, slide-based approaches begin with analyzing patches as patch-wise analyses do, but combine the analysis results of the patches to make a single prediction. Slide-based approaches are mainly bifold: (1) post-hoc aggregation and (2) features aggregation. First, a post-hoc aggregation performs patch-wise analyses across a WSI and combines the predictive scores of the patches to compute a slide-based score, where its key algorithm is a strategy how to combine the patch-wise outcomes. The highest value of patch-wise probability scores was considered as a slide-based probability score^17^. The average of the probability scores, which were generated by multiple pretrained models in a patch, was computed as a confidence probability score on each patch. Then, the weighted mean value of the top-*K* patch confidence probability scores was considered as a slide-based probability score^18^. Important patches were identified by the expectation-maximization algorithm, and the histograms of the patch scores were introduced to a Support Vector Machine (SVM) for computing a slide-based outcome^19^. Patch-wise probability scores generated a frequency count matrix, and a logistic regression was trained on the matrix for whole slide-based lung cancer diagnosis^20^. Majority voting on the patch-wise probability scores classified cancer or normal on radiology and pathology slides^21^.

Second, the feature aggregation approach identifies clinically associated morphological features from patches using a pretrained model, and then aggregates the features to make a slide-based prediction. Feature extraction from patches and aggregation play an important role in the feature aggregation approach. For instance, predesignated phenotype features, such as cell count, size, and density, aggregated as slide-level features using simple aggregation for cancer classification^17^, DNA repair deficiency^22^, and HER2 protein fusion analysis^23^. Statistical features, such as mean, median, and variance of patch-wise predictive scores, were introduced to random forest for lung cancer sub-type classification^24^. Intermediate outcomes of patches were obtained as patch features, and they were combined to predict slide-based outcomes of a patient^25–27^. A grid-based feature extraction was performed on entire WSI, where attention maps were generated by aggregating grid features as slide-based features^28^. The activation values in the penultimate layer of ResNet50 were considered as a low-dimensional topology of patch images, and *k*-nearest neighbors was applied to generate adjacency graphs couple with graph CNNs^29^. Multi-instance learning as feature extraction coupled with Recurrent Neural Networks (RNN)^30^ and transformer-based multi-layer perceptron^31^ as feature aggregation were used for slide-based analysis, in which they considered only top-*k* probability patches for slide-based classification. However, feature extraction in both post-hoc and feature aggregation approaches often requires prior domain knowledge to define features (e.g. cell shape), which are task-specific, rather than generalized frameworks for whole-slide image analysis.

Whole slide histopathology image analysis has often leveraged weakly supervised learning, due to the limited availability or high cost of strongly supervised data with pixel-wise ROI annotation. In weakly supervised learning, image-level labels indicate only clinical categories or status of a pathological image without the location information associated with the image-level labels. Thus, all patches are typically assigned the same label of the slide in weakly supervised learning, which creates false positive samples (e.g., non-cancerous regions in a cancer patient’s sample)^32^. Then, weakly supervised learning localizes regions of interest by excluding the false positive patches. Most weakly supervised learning studies often rank patches with class-specific morphological features to reduce the impacts from the false positive patches^33^. For instance, whole slide-image segmentation was performed to recognize the false positive regions by iteratively penalizing patches of low scores^34^. Patches with high loss scores were excluded from training data for the cancer localization^35^. Patches coarsely annotated by pathologists were weighted to improve the learning performance^17^. Regions with uncertain prediction were identified using min-max uncertainty regularization and constrained to reduce the impact of false positive regions in weakly supervised learning^36^.

In the weakly supervised learning, Class Activation Maps (CAM) have been widely used for automatic class-specific feature extraction and visualization to identify regions of interest^37–39^. In general, CAM are generated from CNN activation to localize class-specific regions from original image^40^. Thus, CAM are used to generate pseudo pixel-wise annotations in weakly supervised learning^41^. The advanced version of CAM, Gradient-CAM (Grad-CAM), localized high risk Melanoma regions to classify Melanoma^42^. CAM at a lower magnification (e.g., 5$\times$) in a WSI were used to identify class-specific regions in a higher magnification (e.g., 20$\times$). Furthermore, the class-specific regions were considered for model training to reduce false positives^43^. CAM in autoencoder identified foreground objects in histopathology images^44^.

**Figure 2:**
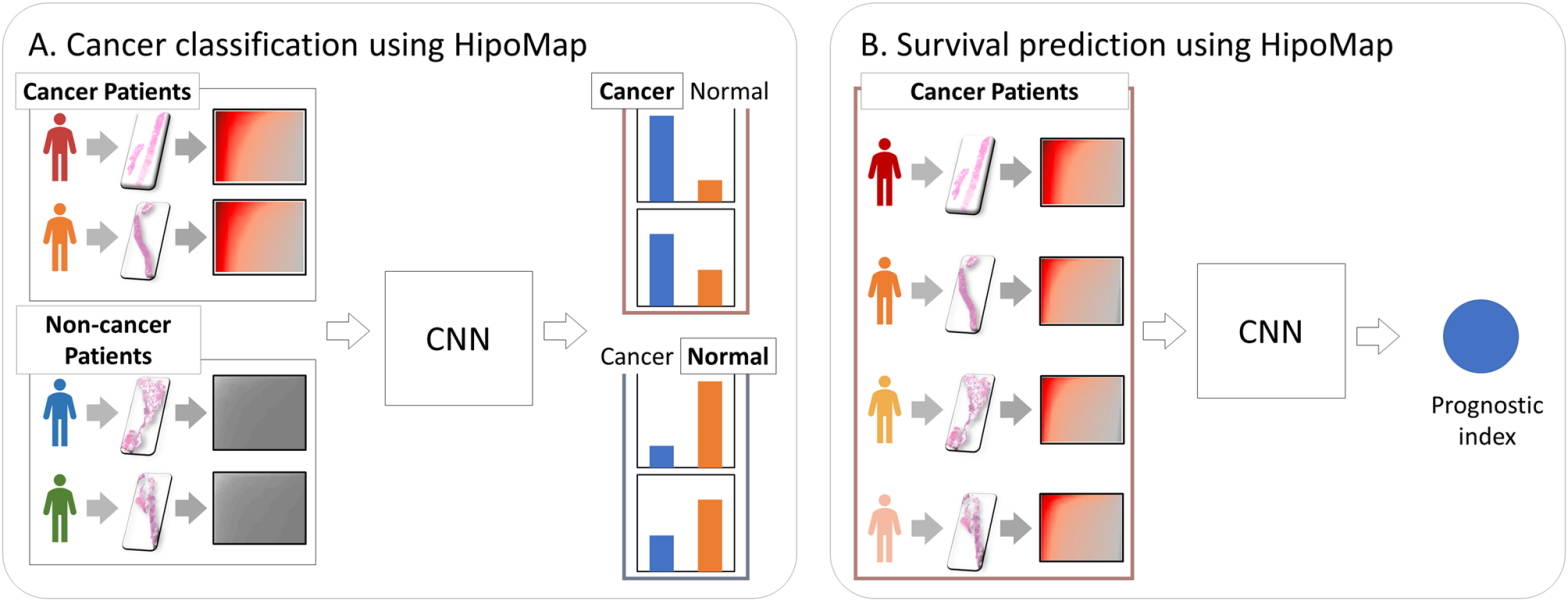
Applications of slide-based analysis using HipoMap but not limited to: (**A**) cancer classification and (**B**) survival analysis.

**Figure 3:**
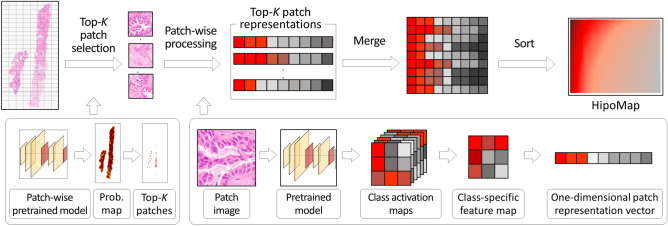
Overview of HipoMap. The proposed method produces a HipoMap that represents a whole slide image through (1) top-*K* patch selection, (2) patch-wise representation, and (3) patch aggregation.

A generalized task-independent framework for slide-based analysis is essential, since most state-of-the-art slide-based analysis methods are task-dependent, which require to develop new strategies or fine-tuning for new applications (e.g., rare disease classification). We propose a novel slide representation of whole slide images, named HipoMap, which is a flexible solution to apply for any slide-based problems (see “Methods”). HipoMap converts a WSI of various shapes and sizes into an image-formatted representation of fixed-size, so that the structured representation can be further analyzed coupled with a deep learning model for slide-based analyses (see Fig. 2). Main contributions of our proposed model HipoMap include: creating a histopathology representation map for various slide-based WSI problems (e.g., survival analysis and subtype classification); efficient training of the model without pixel-wise ROI annotations; and outperforming the current state-of-the-art methods in slide-based pathological image analysis. Specifically, HipoMap consists of the following steps, (1) top-*k* patches selection, (2) patch representation, and (3) patch aggregation (see Fig. 3). First, top-*K* patches are identified by patch probability to be robust to noises and outlier patches. Second, Grad-CAM produces a one-dimensional class representation vector from each patch. Finally, the one-dimensional class representations are aggregated to generate an image-formatted matrix (HipoMap), which provides a flexible solution to apply any advanced machine learning methods, such as CNN, for slide-based analysis in various applications .

## Results

We assessed HipoMap with various settings and datasets for cancer classification, subtype classification, survival analysis, and survival prediction, which are representative applications of slide-based analysis, so that we verify the performance of HipoMap in various applications and prove that HipoMap is task-independent. We, first, conducted experiments for the slide-based cancer classification and subtype classification using lung cancer biopsies obtained from Gyeongsang National University Hospital (GNUH). Then, we compared the performance of survival analysis and survival prediction with state-of-the-art methods using Lung Cancer Adenocarcinoma (TCGA-LUAD) at The Cancer Genome Atlas (TCGA) repository. We further analyzed HipoMap with Stomach Adenocarcinoma (TCGA-STAD) and Colon Adenocarcinoma (TCGA-COAD) datasets. The datasets used in this study are described in Table 1.

### Cancer classification using the GNUH lung cancer dataset

We conducted experiments with Hematoxylin and Eosin (H &E) stained pathology slides, obtained from 102 cases with lung or bronchus biopsies in 2012 and an additional 11 patients diagnosed with large cell neuroendocrine carcinoma (LCNEC) in the biopsy in between 2012 to 2018 at Gyeongsang National University Hospital in Korea. Among the pathology slides, 19, 18, and 18 cases were diagnosed as squamous cell carcinoma (SCC), adenocarcinoma (ADC), and small cell lung carcinoma (SCLC), respectively, and the others (n=47) were non-tumor cases. The diagnoses were histopathologically confirmed by two experienced pathologists. Then, the 113 digital WSIs were acquired from the pathology slides with the Aperio AT2 slide scanner (Leica Biosystems Division of Leica Microsystems Inc., IL, USA) at 40x magnification levels. This study was approved by the Institutional Review Board of Gyeongsang National University Hospital with a waiver for informed consent (2021-04-016). The preprocessing and patch extraction were performed with the open-source python package, PyHistopathology (http://dataxlab.org/pyhistopathology). We applied a naive color normalization with a reference WSI of high-quality^45^ to focus on assessment of our general framework for slide-based analysis. Note that there are a number of advanced techniques of color normalization for pathological image analysis^46^, such as wavelet decomposition^47^, clusters centroid^48^, sparse autoencoders^49^, and generative adversarial network^50^. Noises and artifacts, including tissue tears, folding, and over-staining, in the WSIs were removed by Gaussian blur smoothing, and the background was filtered out by thresholding. Patches containing at least 20% tissues were considered for this study.

**Table 1:** Dataset description for experiments on several applications of slide-based analysis.

Experiments	Dataset	Description
Cancer classification	GNUH	tumor (N=66) vs non-tumor (N=47)
Subtype classification	GNUH	SCC (N=19), ADC (N=18), SCLC (N=18), LCNEC (N=11)
Survival analysis and prediction	TCGA-LUAD	Censored (N=357), uncensored (N=150)
Survival prediction	TCGA-LUAD	Uncensored (N=150)

**Table 2:** Slide-based AUCs of the HipoMap with various top-*K* values on the validation data with the pretrained models of CAT-Net (left) and GB (right). The highest AUCs are highlighted in bold.

Model	Top K	AUC	Model	Top K	AUC
HipoMap with CAT-Net	K=25	0.948±0.023	HipoMap with GB	K=25	0.932±0.027
	**K=50**	**0.973**±**0.014**		**K=50**	**0.954**±**0.013**
	K=100	0.971±0.016		K=100	0.952±0.018
	K=150	0.959±0.026		K=150	0.942±0.028

**Table 3:** Comparison of AUC between the benchmark methods and our proposed HipoMap. The highest AUCs are highlighted in bold.

Pretrained model	Method	AUC	Pretrained model	Method	AUC
CAT-Net	Logistic	0.778±0.082	GB	Logistic	0.752±0.093
	Mean	0.745±0.091		Mean	0.734±0.089
	HMRF	0.763±0.063		HMRF	0.716±0.057
	Histo-SVM	0.811±0.037		Histo-SVM	0.805±0.029
	ATSA	0.885±0.017		ATSA	0.855±0.023
	RNNSA	0.915±0.014		RNNSA	0.891±0.017
	**HipoMap**	**0.966**±**0.028**		**HipoMap**	**0.944**±**0.035**

The entire WSIs were randomly split into training (75%) and test data (25%) using stratified sampling, due to the small number of test samples for LCNEC. Then, the training data was further proportionally split into training (80%) and validation (20%). On each experiment, 28, 7, and 12 slides of normal and 38, 10, and 16 of cancer slides were considered for training, validation, and test, respectively. We extracted non-overlapping patches of 299 $\times$ 299 pixels at the 20$\times$ magnification level. Finally, we obtained approximately 160,000 patches from the cancer slides and 140,000 patches from the non-tumor slides. We repeated the experiments ten times for reproducibility, where we trained the pretrained models using the training data for each experiment.

We compared the performance of our proposed method with six state-of-art methods designed for WSI classification. The benchmark methods included: (1) Logistic regression coupled with CNNs (a.k.a. Logistic)^20^, (2) Mean of patch scores (a.k.a. Mean)^18^, (3) a Heat Map-based Random-Forest Classifier (a.k.a. HMRF)^24^, (4) Histogram-based iterative SVM (Histo-SVM)^19^, (5) Attention pooling-based slide analysis (ATSA)^31^, and (6) RNN-based slide analysis (a.k.a RNNSA)^30^. Logistic, HMRF, ATSA, and RNNSA are feature aggregation approaches, whereas Mean and Histo-SVM are post-hoc aggregation approaches. Note that we used the same patch-wise pretrained model on the all benchmark models in the experiments to compare the performance.

For the implementation of Logistic, confusion matrices were generated from patches, and logistic regression was applied to the confusion matrices for classifying a WSI using the scikit-learn library in Python. For Mean, a mean value of patch probability scores was considered as a score of a slide. The original paper introduced a confidence-based voting strategy for a multi-class classification problem, whereas we modified the method to a binary classifier to compare with HipoMap^18^. For HMRF, probability maps were constructed from patch probability scores using pre-train models. Fifty statistical and morphological features were extracted from probability maps, and the extracted features were introduced to a random forest classifier for the slide-based analysis. We used OpenCV and scikit-learn libraries for the feature extraction and random forest classifier respectively. For Histo-SVM, a histogram of patch probability was extracted and introduced to SVM for slide-based scores. For ATSA and RNNSA, the original papers used Multi instance learning (MIL) for patch-wise training, whereas we considered pretrained models of CAT-Net and GB. For all methods, the hyper-parameters of the learning rate and L2-norm regularization were obtained to optimize the validation data using a grid search^51^. HipoMap was implemented in Keras with TensorFlow as back-end. The performances of HipoMap with various top-patch size *K* were evaluated (Table 2). A lower value of the hyperparameter *K* may cause missing some important patches to conduct the slide-based analysis, whereas a higher value of *K* may include more false positive patches in the representation map. We used *K*=50, which showed the best performance on the validation, for all experiments in this paper.

For the comparison, we considered two pretrained CNN models with patches of size 299$\times$299 as a backbone model: (1) Cancer-Texture Network (CAT-Net)^13^ and (2) Google Brain (GB)^12^. CAT-Net and GB were trained with the training data to classify cancer and normal slides with patches of size 299$\times$299. For the pretrained CNN models, ROI annotations were not available. The optimal hyper-parameters, such as learning rate, dropout, weight-decay, and optimizer (e.g., SGD, ADAM), were obtained using a grid search on the validation data with minimum validation loss.

**Figure 4:**
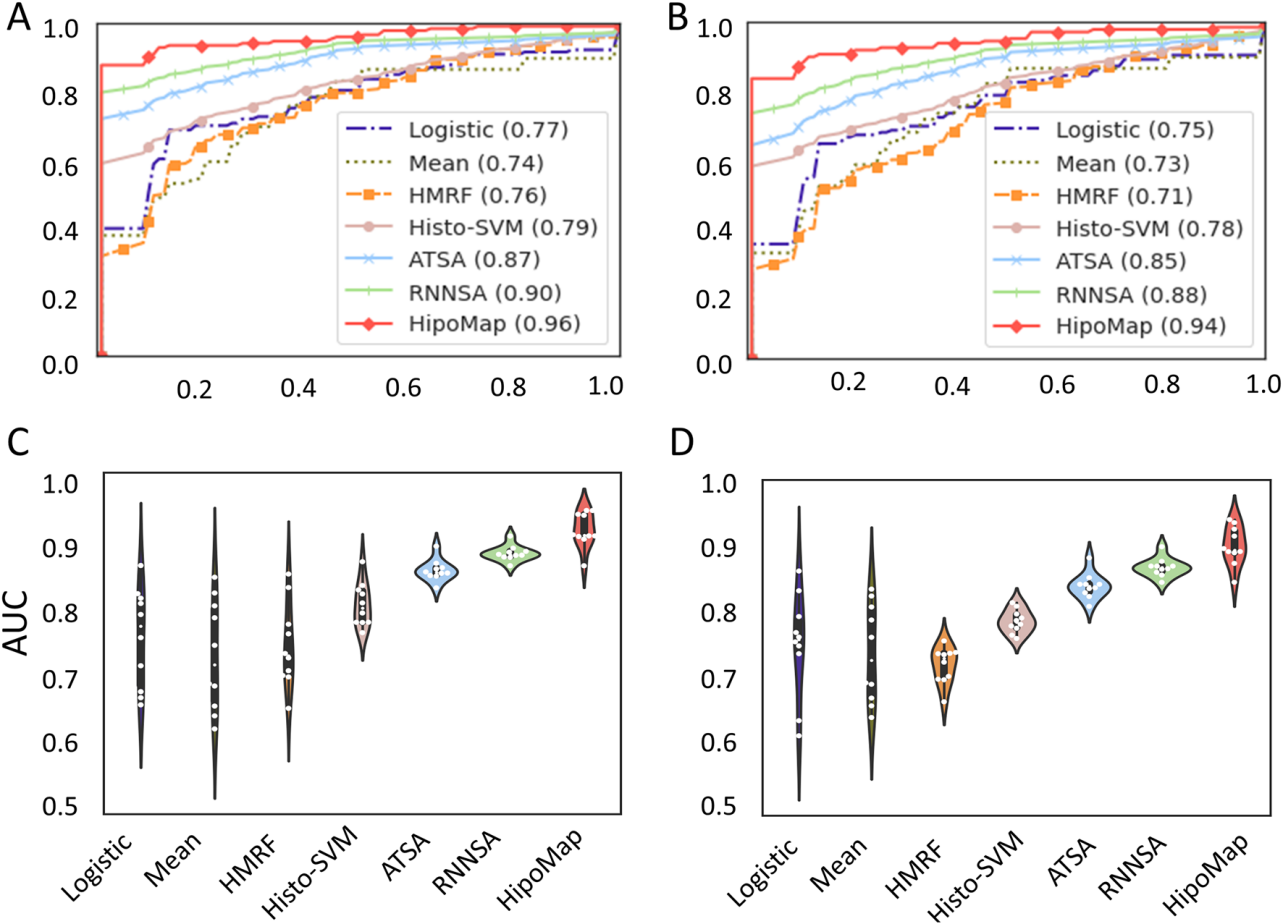
Overall slide-based performance of the benchmark methods and our proposed HipoMap: (**A**) ROC with CAT-Net, (**B**) ROC with GB, (**C**) AUCs with CAT-Net, and (**D**) AUCs with GB.

We measured the Area Under the Curve (AUC) on the test data. The experiments showed that HipoMap outperformed the other six benchmark methods (Table 3 and Fig. 4). HipoMap produced the best AUC of $0.966\pm 0.026$ and $0.945\pm 0.034$ with the pretrained models of CAT-Net and GB respectively, whereas the second highest AUCs were $0.915\pm 0.014$ and $0.891\pm 0.017$ with RNNSA. HipoMap showed at least 5% more improvements on AUC than the others.

**Figure 5:**
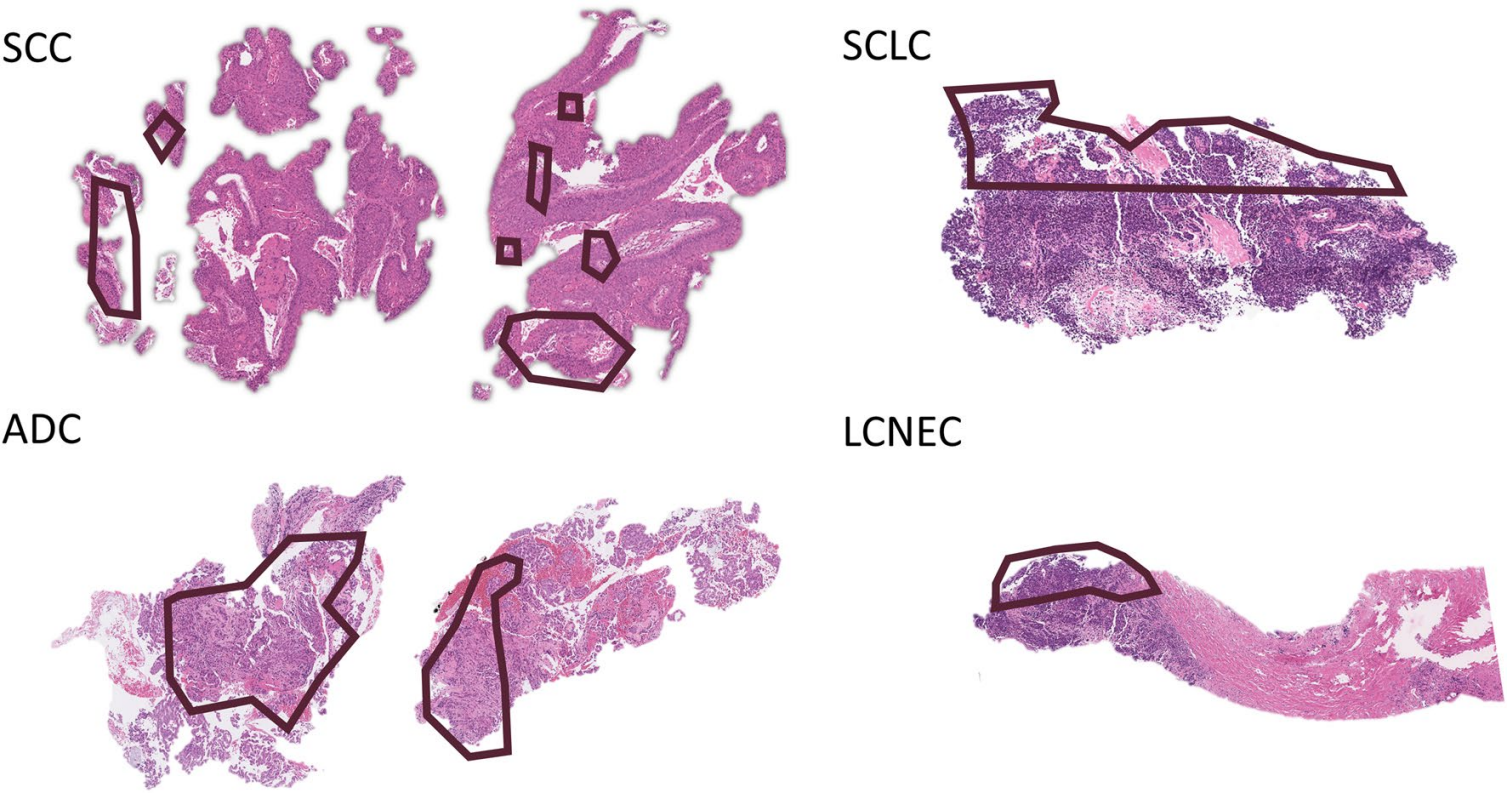
Top *K*-patches in WSI. Top-50 patches are annotated.

We further investigated pathological characteristics of the top-*K* patches. It is critical that the top-ranked patches include discriminative patterns to perform a slide-based task, since HipoMap examines only the top-*K* patches. In the slide-based cancer classification experiments using the GNUH data, the top-ranked patches with high cancer probabilities included the most cancerous regions, such as cancer cells and peri- and intratumoral stromal tissues, in most slides. On the other hand, the regions of inflammation and fibrosis on non-tumor slides showed low or intermediate values in the probability maps, which were ranked low. Interestingly, we found different patterns of the top-ranked patches between the SCC/ADC and SCLC/LCNEC slides. The top-50 patch images in SCC and ADC slides were mainly distributed in the peri- and intratumoral stromal tissues (see SCC and ADC in Fig. 5). On the other hands, the top-50 patch images of SCLC and LCNEC were distributed in the cancer cells (see SCLC and LCNEC in Fig. 5). Although it is not pathologically clear why the peri- and intratumoral stromal tissues showed a higher cancer probability in SCC and ADC, one possible explanation is that the peri- and intratumoral stromal tissues may distinguish from non-cancer-related stromal tissues in the given slides.

**Figure 6:**
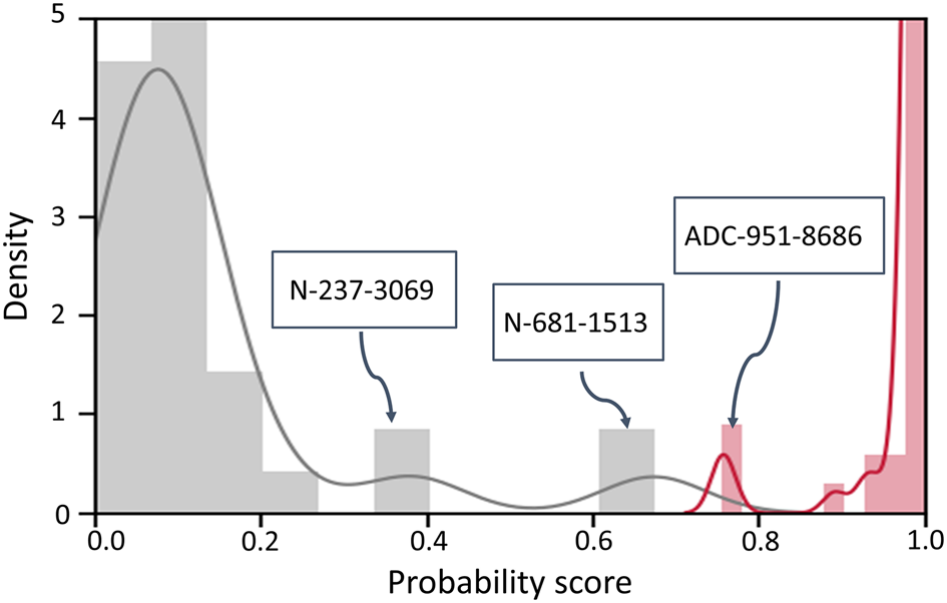
Slide-based cancer probabilities of the three samples, N-S12-13682, N-S12-15479, and ADC-S12-13080, among the distribution of the entire GNUH lung cancer dataset by HipoMap. The distribution of the normal slides is in gray, whereas the probabilities of cancer are in red.

**Figure 7:**
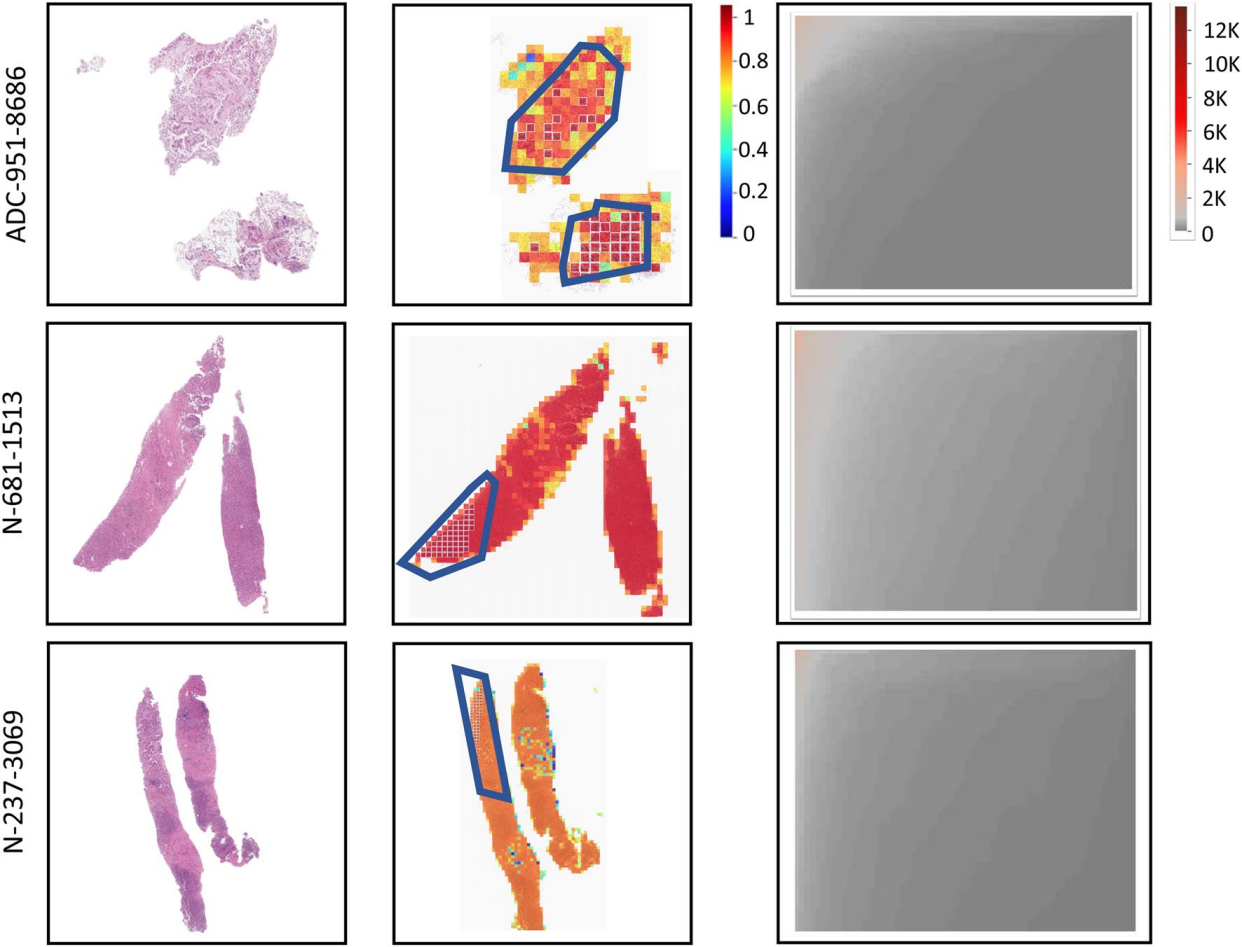
The original WSIs (left), patch-wise probability maps (middle), and HipoMaps (right) of the three samples, N-237-3069, N-681-1513, and ADC-951-8686.

We also examined slides, whose HipoMap scores are on the border of classification (Figs. 6 and 7). One ADC case (sample ID: ADC-951-8686) and two non-tumor slides (sample ID: N-681-1513 and N-237-3069) showed indeterminate HipoMaps scores between 30% and 80%. ADC-951-8686 is well-differentiated, and the proportion of cancer tissue is relatively less than other ADC samples. N-681-1513 contains severe chronic inflammation with fibrosis, which showed high cancer probability. However, another similar slide, N-237-3069, containing severe chronic inflammation with fibrosis showed relatively lower HipoMaps scores and intermediate cancer probability as shown in Fig. 6. Pathologists noted that the two slides of non-tumor patients (N-681-1513 and N-237-3069) have no substantial morphological difference, whereas HipoMap showed the disparity of around 20% on the cancer probability. Fig. 7 showed that both the probability map and HipoMap of N-681-1513 overall includes higher scores than N-237-3069. The supplementary document includes original slide, patch-wise probability map, and HipoMaps of selected GNUH dataset, including N-681-1513, N-237-3069, and ADC-951-8686 (Figs. S1–S11).

### Subtype classification

**Figure 8:**
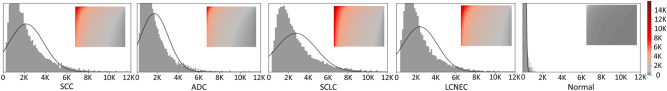
The histograms of pixel values on the averaged HipoMaps of the four subtypes of lung cancer and non-tumor patients, and the averaged HipoMaps (top right corner) on each group.

**Figure 9:**
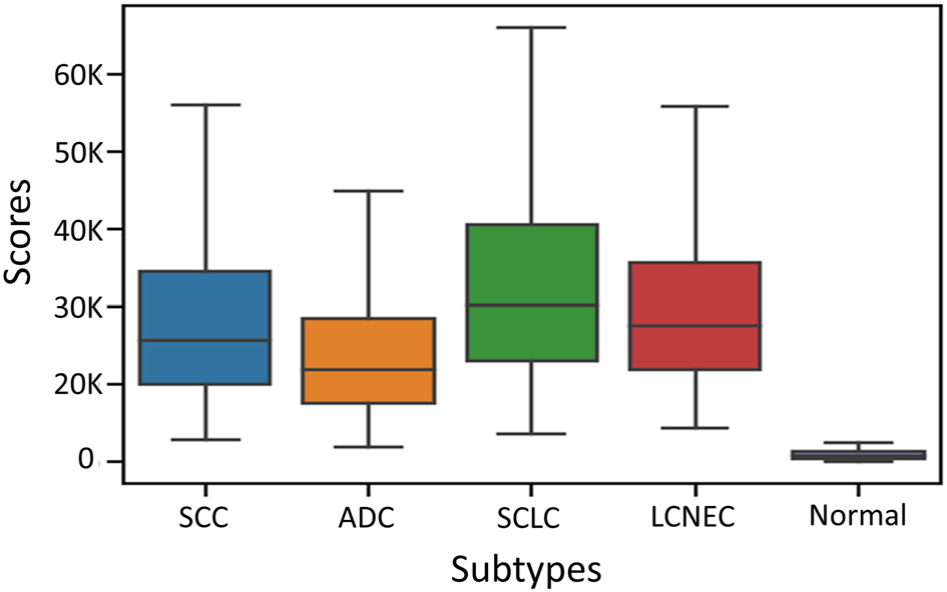
HipoMap distributions on the subtypes.

We compared the HipoMaps of the four subtypes of lung cancers (i.e., SCC, ADC, SCLC, and LCNEC) to verify that patterns of HipoMap are aligned with well-known pathological knowledge of subtypes. The subtypes are determined by pathological morphologies in WSIs, so their HipoMap representation should reflect the differences. First, we generated HipoMaps for all WSIs, each of which produced a HipoMap using a pretrained model trained by the training data that does not belong to the WSI. Then, the HipoMaps were averaged on each subtype group and the non-tumor patient group. Figure 8 depicts histograms of pixel values on the HipoMaps along with the averaged HipoMap (top right corner) on each group, while Fig. 9 illustrates the distribution of the pixel values as a boxplot. In the experiment, we considered only HipoMap with CAT-Net, which showed the best performance. The overall values on the averaged HipoMaps of SCC and ADC show lower than SCLC and LCNEC in Fig. 8. It may be because SCC or ADC can be well-differentiated and similar to benign or reactive lesions, such as squamous metaplasia and type 2 pneumocyte hyperplasia. On the other hand, SCLC and LCNEC are high grade neuroendocrine carcinomas and poorly-differentiated, so these carcinomas morphological patterns are more distinguishable from normal or reactive cells. Therefore, SCLC and LCNEC may show higher average scores because reactive counterpart lesions for these carcinoma can be hardly found in non-tumor slides.

**Table 4:** Slide-based micro- and macro-F1 score of HipoMaps with various subtypes of lung cancer with the pretrained models of CAT-Net (left) and GB (right). The highest micro- and macro-F1 scores are highlighted in bold.

Pretrained model	Method	Micro F1	Macro F1	Pretrained model	Method	Micro F1	Macro F1
CAT-Net	HMRF	0.523±0.094	0.673±0.087	GB	HMRF	0.528±0.084	0.654±0.081
	Histo-SVM	0.541±0.098	0.647±0.068		Histo-SVM	0.505±0.092	0.613±0.086
	ATSA	0.657±0.056	0.721±0.045		ATSA	0.644±0.047	0.725±0.067
	RNNSA	0.683±0.044	0.754±0.053		RNNSA	0.671±0.053	0.742±0.048
	**HipoMap**	**0.704**±**0.051**	**0.773**±**0.046**		**HipoMap**	**0.685**±**0.046**	**0.752**±**0.053**

Furthermore, we compared the performance to classify lung cancer WSIs subtype as a multi-class classification problem. The benchmarks of HMRF, Histo-SVM, ATSA, RNNSA were extended to multi-class classifiers, but the binary classifiers of Logistic, Mean were not included in this experiment. HipoMap achieved the highest micro- and macro-F1 scores of 0.704±0.051 and 0.773±0.046 respectively (Table 4).

### Survival analysis using TCGA datasets

We verified HipoMap’s performance for survival analysis using publicly available TCGA repository. We applied HipoMap for 507 WSIs, including censoring data, in Lung Cancer Adenocarcinoma (TCGA-LUAD). We considered diagnostic WSIs only. We generated HipoMaps of the entire TCGA-LUAD WSIs with the pretrained model of CAT-Net and GB, which were trained with the GNUH lung cancer data. Note that HipoMap captures comprehensive morphological patterns of cancer, whereas the simple CNN trains to predict survivals. We performed 5-fold cross-validation on the entire samples, where we used 20% of the training data as validation data. We trained the simple CNN model with the training data of TCGA-LUAD, where the CNN model used a linear activation in the output layer to generate prognostic index for survival analysis. We used negative log likelihood as a loss function of Cox regression model and SGD as an optimizer.

**Figure 10:**
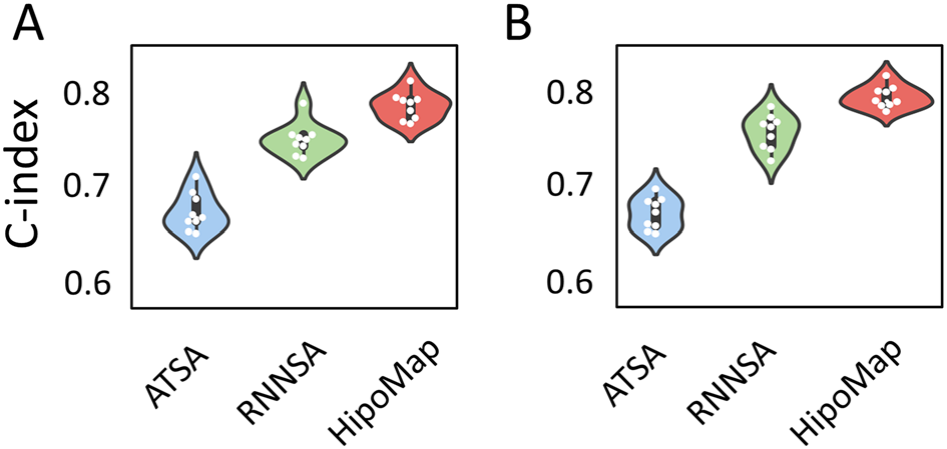
C-index with (**A**) CAT-Net and (**B**) GB in the benchmark methods and our proposed HipoMap for survival analysis.

We considered the negative log likelihood as loss function for ATSA and RNNSA. We did not consider the benchmark method, Mean, for this experiment, because it is designed for classification problems. The optimal hyper-parameters were obtained using a grid search on the validation data with minimum validation loss. We computed c-index as an evaluation metric for survival analysis, and we repeated the experiments ten times. Table 5 and Fig. 10 show HipoMap’s outperformance in survival analysis, compared to the benchmark methods. HipoMap achieved the highest c-index of 0.787 ± 0.013, and 0.763 ± 0.016 with pretrained models of CAT-Net and GB respectively, which was 4.7% improved compared to second highest benchmark of RNNSA.

**Table 5:** Comparison of c-index between the benchmark methods and our proposed HipoMap. The best performance is highlighted in bold.

Pretrained model	Method	C-index	Pretrained model	Method	C-index
CAT-Net	ATSA	0.674±0.019	GB	ATSA	0.642±0.015
	RNNSA	0.752±0.016		RNNSA	0.726±0.018
	**HipoMap**	**0.787**±**0.013**		**HipoMap**	**0.763**±**0.010**

### Survival prediction on uncensored TCGA dataset

**Figure 11:**
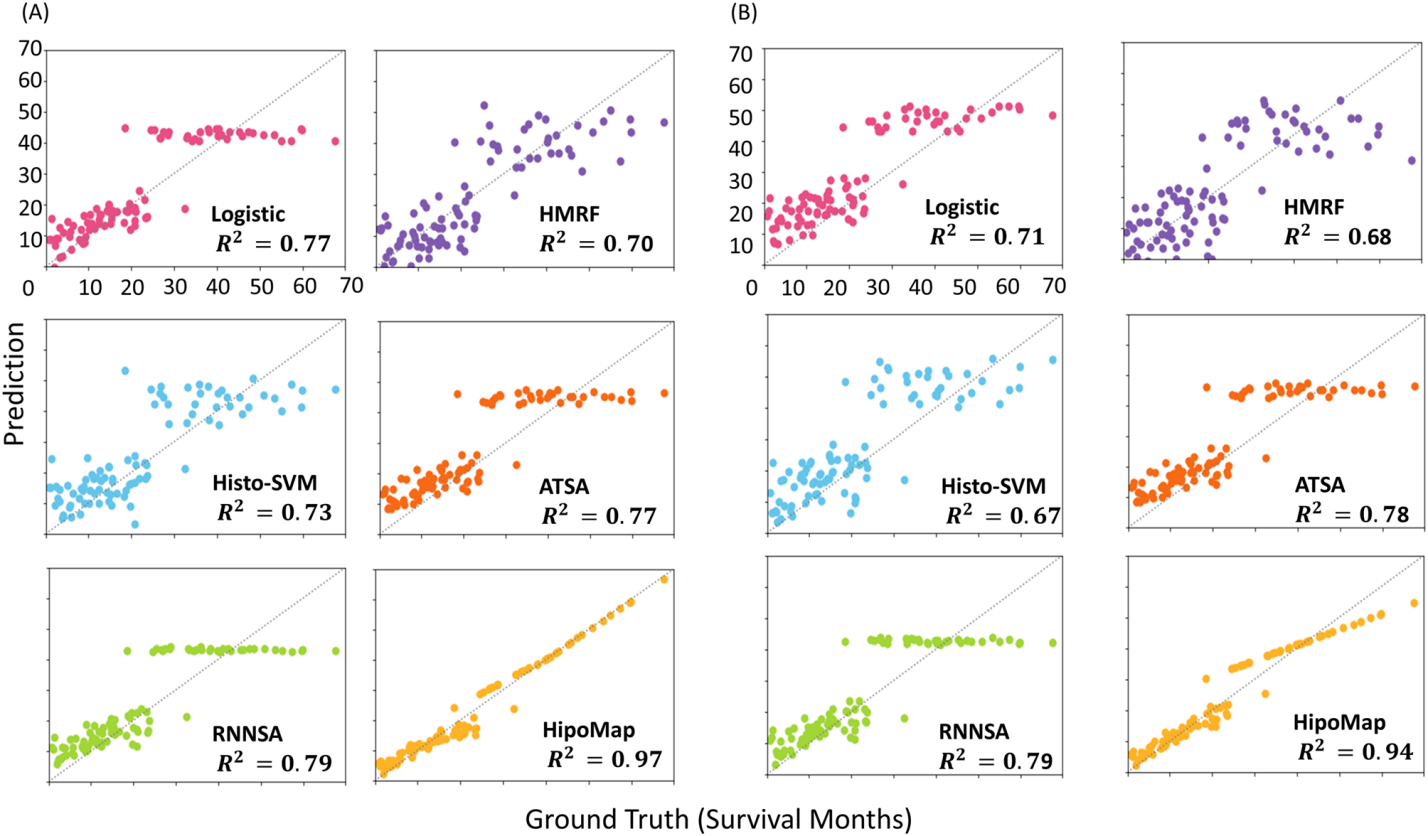
Survival prediction using HipoMap on 150 whole slide images in TCGA LUAD. (**A**) Results with CAT-Net as pretrain and (**B**) Results with GB as pretrain.

**Figure 12:**
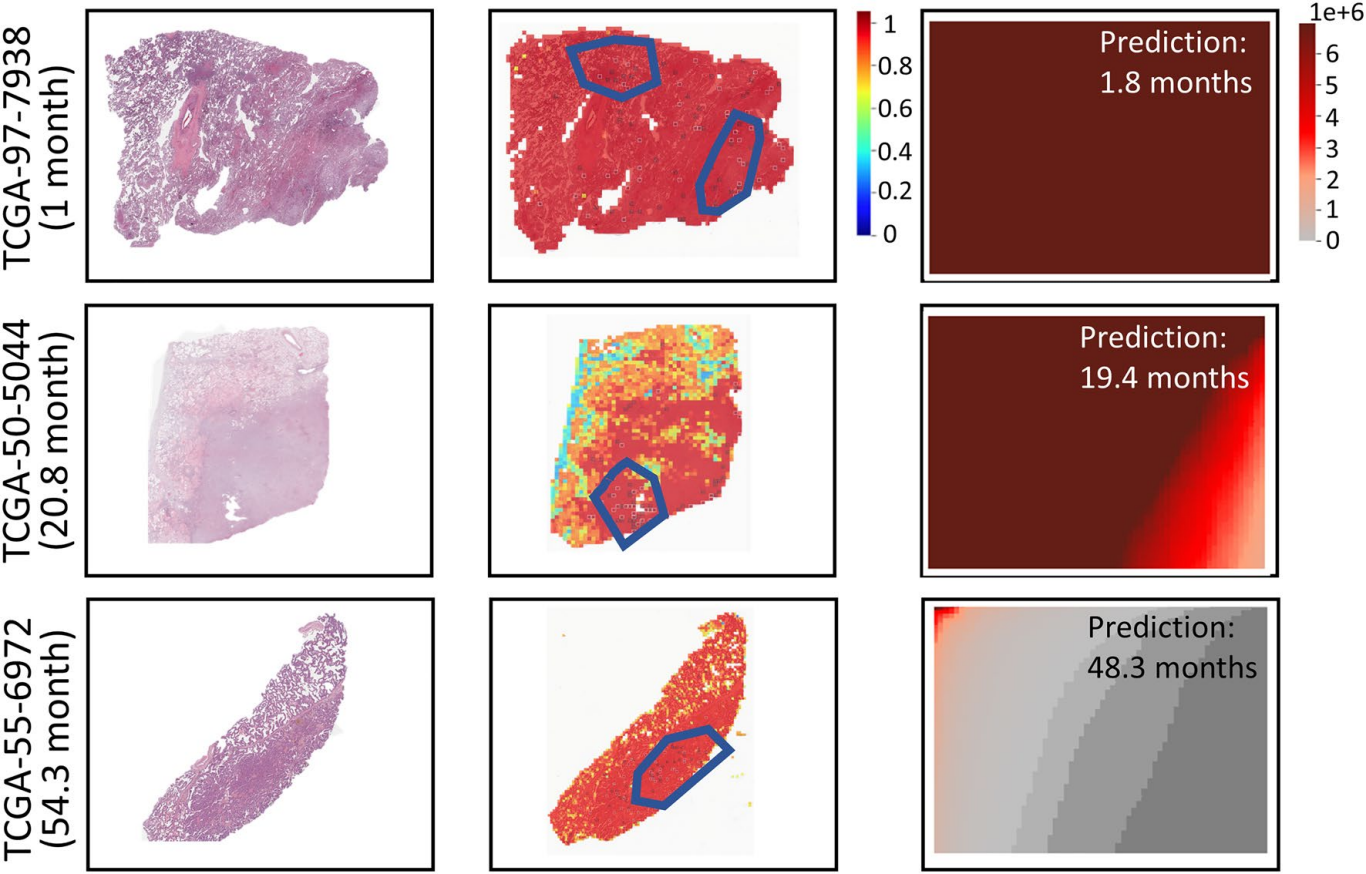
The original WSIs (left), patch-wise probability maps (middle), and HipoMaps (right) of the three patients, who survived one month (TCGA-97-7938), 20.8 months (TCGA-50-5044), and 54.3 months (TCGA-55-6972) in TCGA-LUAD.

Moreover, we tested the performance of HipoMap with uncensored dataset of TCGA Lung Cancer for a regression problem to predict actual survival months. First, we estimated the survival of patients from 150 WSIs (excluding censored data from 507 samples) in TCGA-LUAD. We used the same experimental procedure with survival analysis but used loss function of ordinary least squares (OLS) for the regression problem. For the benchmark, we considered linear regression, random forest regression, SVM regression, ATSA, and RNNSA. We computed Root Mean Square Error (RMSE) and the coefficient of determination ($\mathrm {R^2}$) between predictions and ground truth. Table 6 and Fig. 11 show HipoMap’s outperformance to estimate survival compared to the benchmark methods. The RMSE and $\mathrm {R^2}$ of HipoMap were 2.77±0.36 and 0.978±0.032 respectively We, furthur, demonstrate the process of HipoMap for survival prediction with three patients with lung cancer, who actually survived for one month (Sample ID: TCGA-97-7938), 20.8 months (TCGA-50-5044), and 54.3 months (TCGA-55-6972). Figure 12 illustrates the original WSI (left), patch-wise probability map (middle), and HipoMap (right) of the three patients. The patch-wise probability maps depict cancer probabilities on the patches by the pretrained model of CAT-Net. The HipoMaps in Fig. 12 illustrate the intensity of comprehensive patterns on lung cancer, since the pretrained model was trained for lung cancer classification. The simple CNN was optimized for survival prediction. The survival estimations of the three patients with HipoMaps were 1.8, 19.4, and 48.3 months, while the ground truths were 1 month, 20.8 months, and 54.3 months, respectively. The overall intensity of HipoMaps on the three patients are inversely proportional to the survival.

**Table 6:** Slide-based performance of survival prediction with HipoMap and the benchmark methods. The best performance is highlighted in bold.

Pretrained model	Method	$R^2$	RMSE	Pretrained model	Method	$R^2$	RMSE
CAT-Net	Logistic	0.778±0.042	8.99±0.89	GB	Logistic	0.714±0.053	10.17±0.74
	HMRF	0.705±0.037	10.34±0.64		HMRF	0.687±0.036	10.65±0.84
	Histo-SVM	0.736±0.047	9.77±0.65		Histo-SVM	0.671±0.041	10.93±0.95
	ATSA	0.755±0.052	9.41±0.61		ATSA	0.781±0.045	8.89±0.86
	RNNSA	0.787±0.051	8.806±0.37		RNNSA	0.790±0.035	8.72±0.87
	**HipoMap**	**0.978**±**0.032**	**2.77**±**0.36**		**HipoMap**	**0.948**±**0.041**	**3.74**±**0.44**

### Survival prediction of STAD and COAD

**Figure 13:**
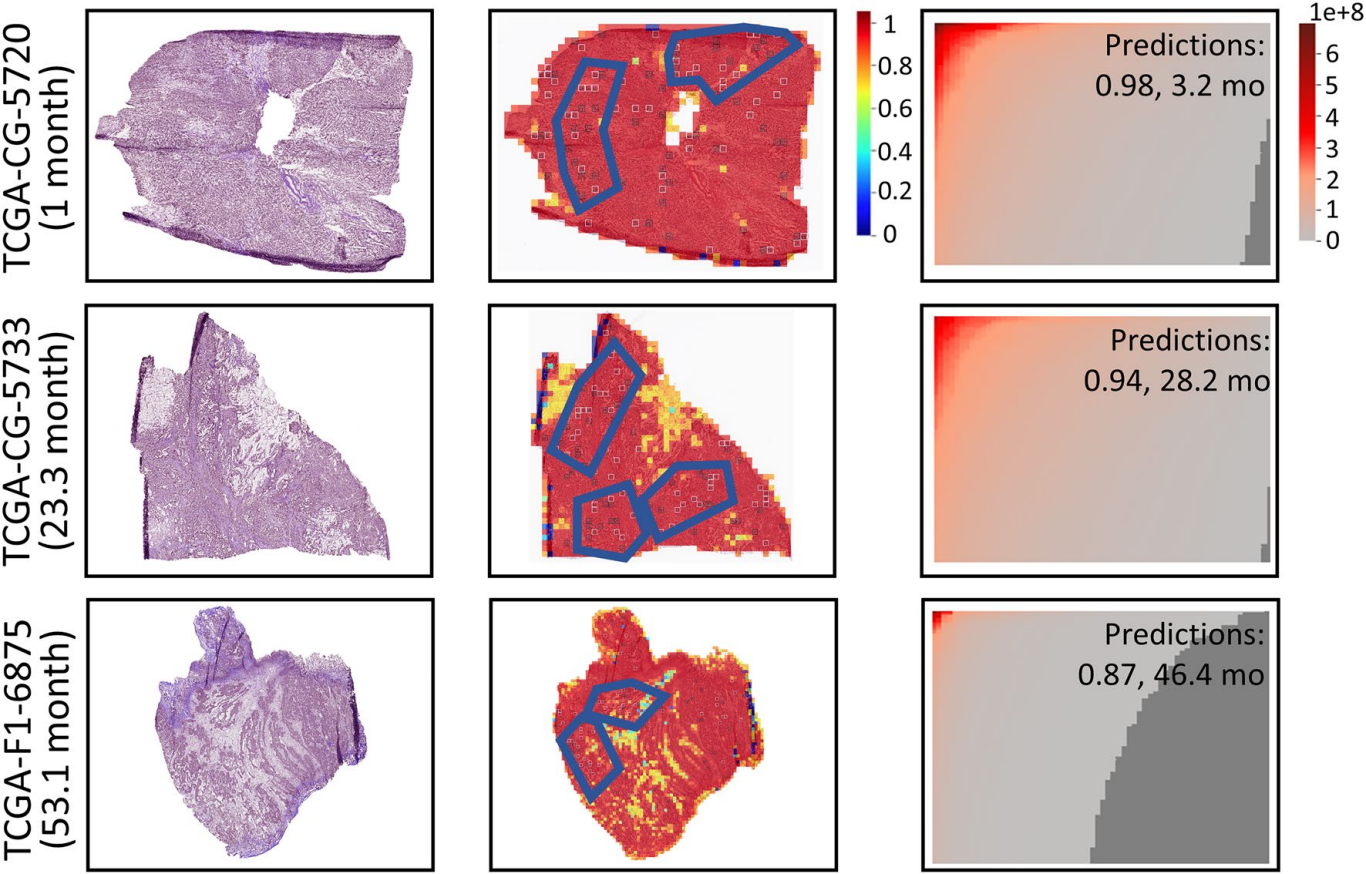
The original WSIs (left), patch-wise probability maps (middle), and HipoMaps (right) of the three patients, who survived one month (TCGA-CG-5720), 23.3 months (TCGA-CG-5733), and 53.1 months (TCGA-F1-6875) in TCGA-STAD.

**Figure 14:**
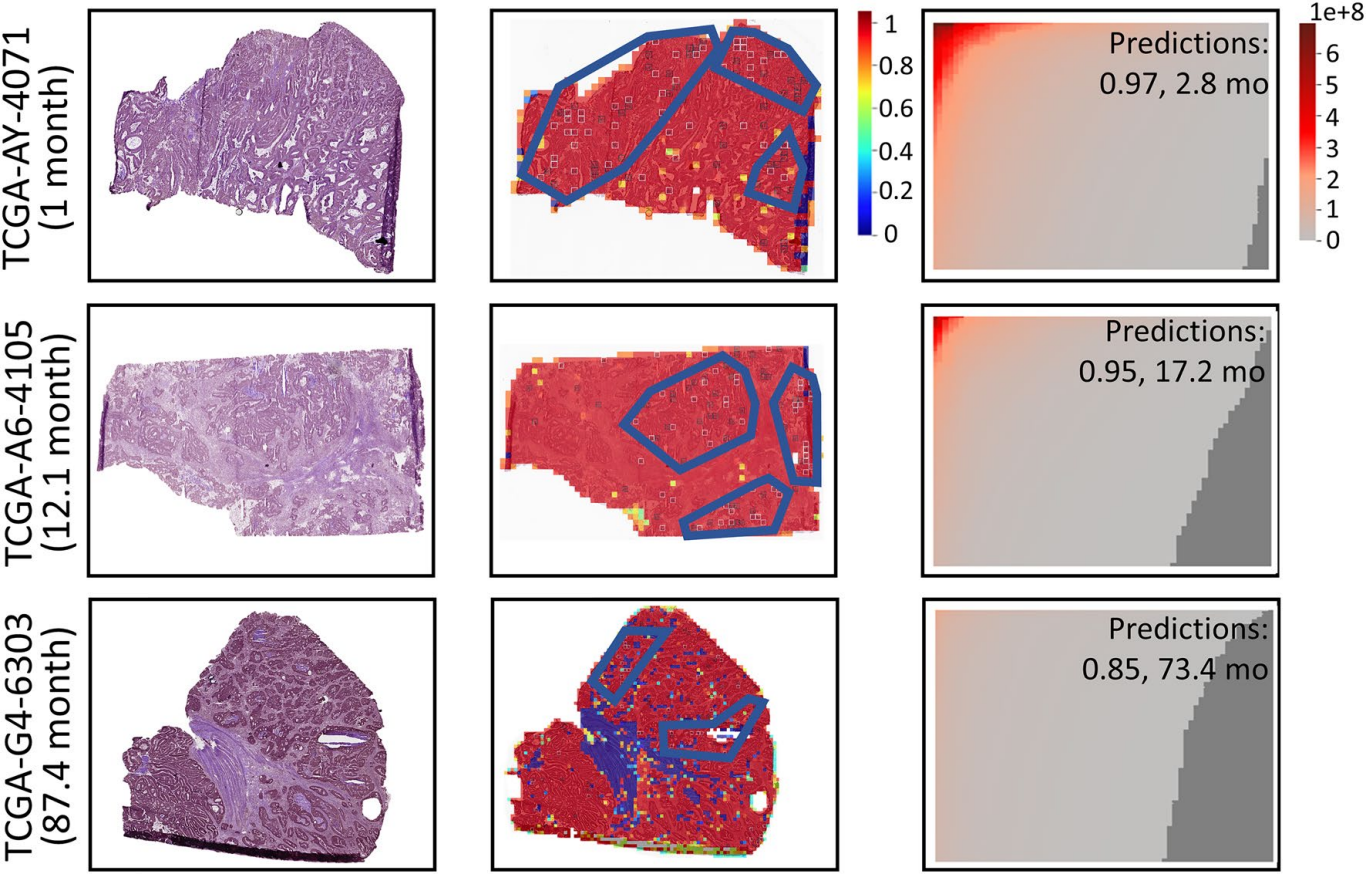
The original WSIs (left), patch-wise probability maps (middle), and HipoMaps (right) of the three patients, who survived one month (TCGA-AY-4071), 12.1 months (TCGA-A6-4105), and 54.3 months (TCGA-G4-6303) in TCGA-COAD.

We applied HipoMap to other TCGA cancers, TCGA-STAD and TCGA-COAD, coupled with the pretrained model (i.e., CAT-Net), which was trained with WSIs in Stomach Adenocarcinoma Carcinoma used in the previous study^16^. The pretrained model was trained by the independent data to the TCGA-STAD or TCGA-COAD. Similarly, we chose three patients on each dataset and examined HipoMap’s patterns with the patients’ survivals. We considered the patients, who survived one month (TCGA-CG-5720), 23.3 months (TCGA-CG-5733), and 53.1 months (TCGA-F1-6875) in TCGA-STAD, and survived one month (TCGA-AY-4071), 12.1 months (TCGA-A6-4105), and 87.4 months (TCGA-G4-6303) in TCGA-COAD. For the samples, HipoMap predicted the survival months of 3.2 months (error = 2.2), 28.2 months (error=4.9), and 46.4 months (error=6.7) in TCGA-STAD, 2.8 months (error = 1.8), 17.2 months (error=5.1), and 73.4 months (error=14) in TCGA-COAD, respectively. The RMSE between the survival predictions and their ground truth was 7.02 months for the six samples. Figures 13 and  14 show the HipoMap’s overall intensities to be reversely proportional to the survival in TCGA-STAD and TCGA-COAD, respectively. Although the high correlation between cancer probabilities and survival is not straightforward evidence to verify the performance on this small number of examples, typically severe cancerous patterns may predict poor prognosis. It may be because HipoMap can be a representation independent to cancer types for slide-based analysis, which may be useful for pan-cancer studies or transfer learning across multiple cancer datasets. For instance, HipoMaps reflect intensities on the patterns of interest in cancer classification or survival predictions, regardless of origins of cancers, through the pretrained models optimized to the corresponding cancer datasets. HipoMap abstracts different morphological patterns on each cancer in a uniform manner. The abstracted representation maps of HipoMap may provide the capability to integrate several cancer datasets easily.

## Conclusion

Automatic whole slide image analysis coupled with advanced machine learning has been changing the paradigm in digital pathology. However, there were few approaches proposed for a slide-based analysis in spite of its many applications. In this study, we developed a novel slide representation of whole slide images, named HipoMap, that can apply to any slide-based problems, such as cancer classification, survival analysis, protein fusion prediction, and protein expression prediction from WSIs. HipoMap converts a WSI of various shapes and sizes to structured image-type representation. HipoMap considers top-*K* patches, which is robust to noises and outlier patches. Grad-CAM in HipoMap take advantages of weakly supervised learning efficiently, where no pixel-based annotation is required. HipoMap efficiently captures morphological dependencies among patches, while analyzing inner morphological patterns in a patch in the same time. The experimental results show that HipoMap is effective than RNN-based methods, since HipoMap considers CNN activation features for prioritizing class-specific morphological patterns, whereas RNN lacks the information because of spatial pooling of features. HipoMap may abstract comprehensive patterns of multiple patches on a whole slide image, as a uniform data structure. HipoMap would be easily extended for pan-cancer studies or transfer learning.

## Methods

In this section, we elucidate our proposed method, HipoMap, for a slide-based analysis. The proposed method consists of the following processes: (1) top-*K* patch selection, (2) patch representation, (3) patch aggregation, and (4) slide-based analysis (Fig. 3). To be short, HipoMap considers top-ranked *K* patches based on predictive scores of a patch-wise pretrained model. Each patch produces a class-specific representation vector. Then, the representation vectors of the top-*K* patches are aggregated to generate an image-formatted matrix, which is called as HipoMap. Finally, HipoMaps are introduced to a CNN to perform a slide-based analysis. The details are elucidated in the following sections.

### Top-*K* patch selection

Shapes and sizes of WSIs are various depending on a biopsy, and class-specific morphology (e.g., cancer cells) may be partially observed in a WSI. HipoMap examines only a fixed number of patches of interest so that all slides produce the same shape of representation maps regardless of the size of WSIs. A predictive score of each patch is computed by a pretrained model. Then, top-*K* patches are selected by ranking the predictive scores, where *K* is a hyper-parameter. For the patches with same predictive scores, a patch with higher averaged activation value is assigned to higher rank. A large *K* may increase the computational cost, whereas a small *K* may reduce the performance of slide-based analysis.

A patch-wise pretrained model is optimized on an objective function of a target task (e.g., classification, survival analysis) using entire patches of the training data, and each patch produces a predictive result (e.g., cancer probability) as a patch-wise analysis. In this study, we used Google-Brain (GB)^12^ and CAncer-Texture Network (CAT-Net)^13^ as pretrained models.

### Patch representations

Each of the top-*K* patches generates a one-dimensional representation vector using Gradients of Class Activation Maps (Grad-CAM) on a patch-wise pretrained model. Grad-CAM identify class-specific morphological patterns of a patch image. Grad-CAM computes importance scores of activation maps (typically in the last convolutional layer), and the weighted averaged activation maps create a class-specific feature map. Specifically, an importance score ($\alpha _m$) for the $m^{th}$ activation map ($1 \le m \le M$) is computed by:

1 $$\begin{aligned} \alpha _m = ReLU\left( \sum _i \sum _j \frac{\partial P_C}{\partial A^m_{ij}}\right) , \end{aligned}$$

where $A^m_{ij}$ is a value in the $i^{th}$ row and the $j^{th}$ column of the activation map ($1\le i\le H, 1 \le j \le W$), and $\partial A^m_{ij}$ is a gradient of the activation function obtained in back-propagation. $P_C$ is an objective function with respect to the target class *C*. Rectified Linear Unit (ReLU) eliminates negative activation maps. In the original paper of GRAD-CAM, ReLU was used to eliminate negative activation values in the class-specific feature map (i.e., the global average of activation maps), whereas we eliminate negative activation maps before generating the class-specific feature map. The class-specific feature map, $T\in \Re ^{H\times W}$, for a patch can be generated by:

2 $$\begin{aligned} T_{ij} = \sum _k^M \alpha _m A^m_{ij}. \end{aligned}$$

The class-specific feature map ($T$) is flattened into the one-dimensional vector and sorted in descending order (i.e., ${\mathscr {F}}\in \Re ^{1\times (H\times W)}$).
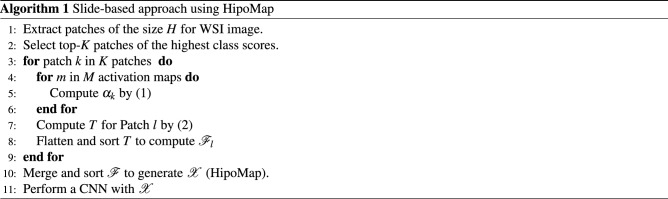


### Patch aggregation

The one-dimensional representation vectors ($\{{\mathscr {F}}_1,\ldots , {\mathscr {F}}_{{K}}\}$) of the top-*K* patches are aggregated to produce a matrix, ${\mathscr {X}}$ $\in \Re ^{{K}\times (H\times W)}$, that represents a whole slide image. The row vectors of the matrix ${\mathscr {X}}$ are sorted by the sums of the row vectors in descending order, so that highly activated patch representations are on the top:

3 $$\begin{aligned} {{\mathscr {X}}}= Sort \Big ( \begin{bmatrix} {\mathscr {F}}_{1},{\mathscr {F}}_{2},\ldots ,{\mathscr {F}}_{{K}} \end{bmatrix}^{\top }\Big ). \end{aligned}$$

### Slide-based analyses

A WSI produces a matrix of fixed-size, HipoMap. For a slide-based analysis, HipoMaps can be analyzed with any machine learning models. In this study, HipoMap is analyzed by a CNN, which is the most popular for imagery data analysis. We used a simple CNN model, which includes three sets of sequential convolutional and max-pooling layers, followed by flattening, fully connected layers, and the output layer. We used 64 kernels of 3 $\times$ 3 for the convolutional layers, and 64 kernels of 2 $\times$ 2 on the average pooling layer. The fully connected layer consisted of 1024 nodes with an activation of ReLU. The hidden layer connected to the output layer with a sigmoid activation for cancer classification and a linear activation for the regression problem of survival estimation. The details of the HipoMap algorithm are in Algorithm 1.

## Supplementary Information

Supplementary Information.

## Data Availability

The Python package is available at https://pypi.org/project/hipomap, and the package can be easily installed using Python PIP. The open-source codes in Python are available at: https://github.com/datax-lab/HipoMap. Supplementary data, including original slides, probability maps, and HipoMapss of the entire GNUH dataset, is available at: http://dataxlab.org/HipoMap/hipomap/SupplyHipoMap.pdf.
