# Supplementary material for: Deep learning-based framework for slide-based histopathological image analysis
Source: Sci Rep. 2022 Nov 9;12:19075. doi: 10.1038/s41598-022-23166-0 (PMC9646838; doi:10.1038/s41598-022-23166-0)
Supplement: Supplementary file 1 — Supplementary Information. [file 41598_2022_23166_MOESM1_ESM.pdf]

	Slide ID	Subtypes (Classes)	Slide-based HipoMap score for cancer classification
Figure S1	SCC-122-6821	Squamous cell carcinoma	0.92
Figure S2	ADC-183-1052	Adenocarcinoma	0.89
Figure S3	SCLC-139-1772	Small cell lung carcinoma	0.96
Figure S4	LCNEC-194-1537	Large cell neuroendocrine carcinoma	0.94
Figure S5	N-155-1721	Normal	0.08
Figure S6	N-155-1552	Normal	0.13
Figure S7	N-154-1322	Normal	0.18
Figure S8	N-155-1465	Normal	0.11
Figure S9	N-681-1513	Normal	0.40
Figure S10	N-237-3069	Normal	0.62
Figure S11	ADC-951-8686	Adenocarcinoma	0.74

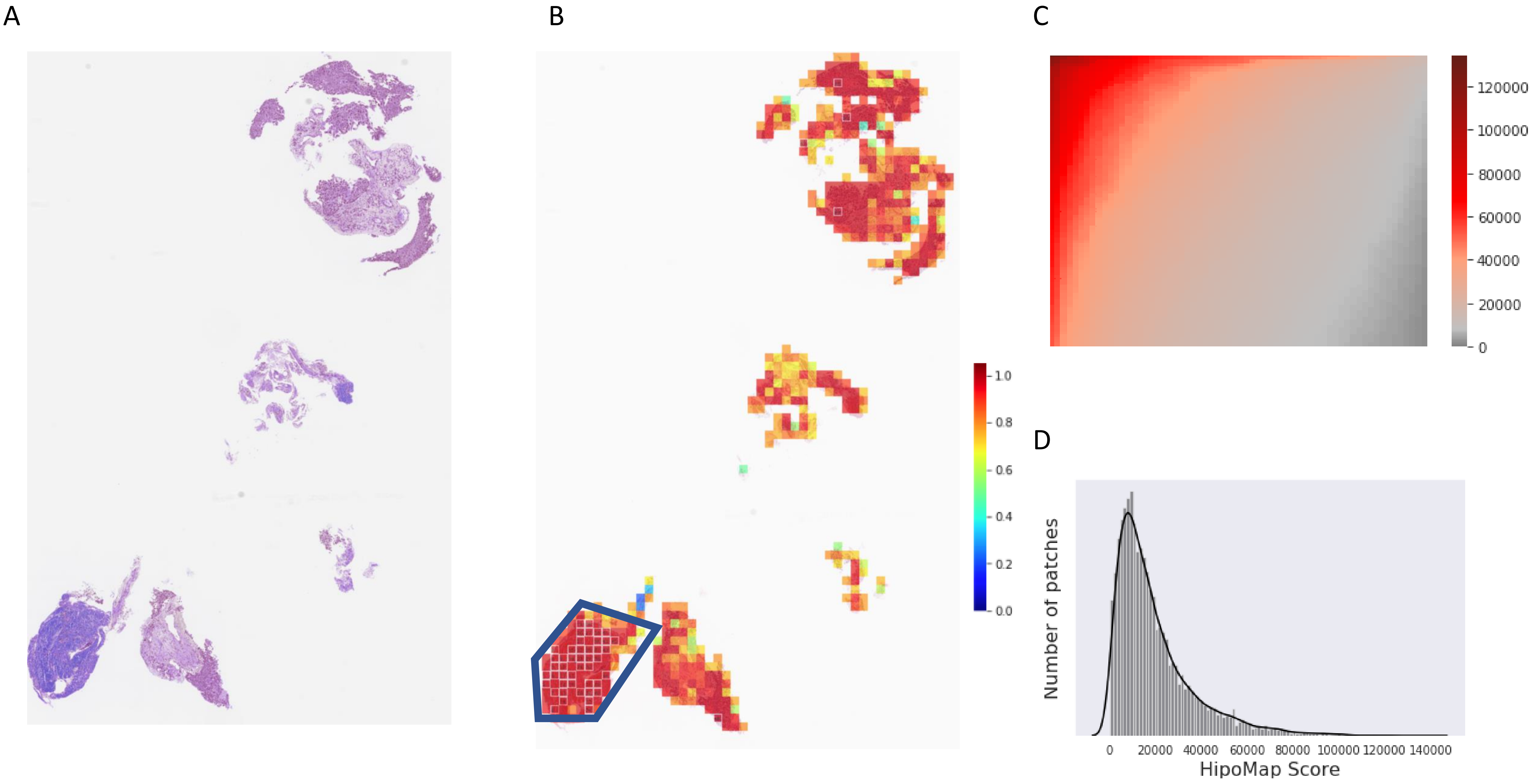

Figure S1. (A) The original WSI, (B) patch-wise probability map, (C) HipoMap, and D) histogram of HipoMap score SCC-122-6821.

*Blue bounding box indicates top 50 probability score patches

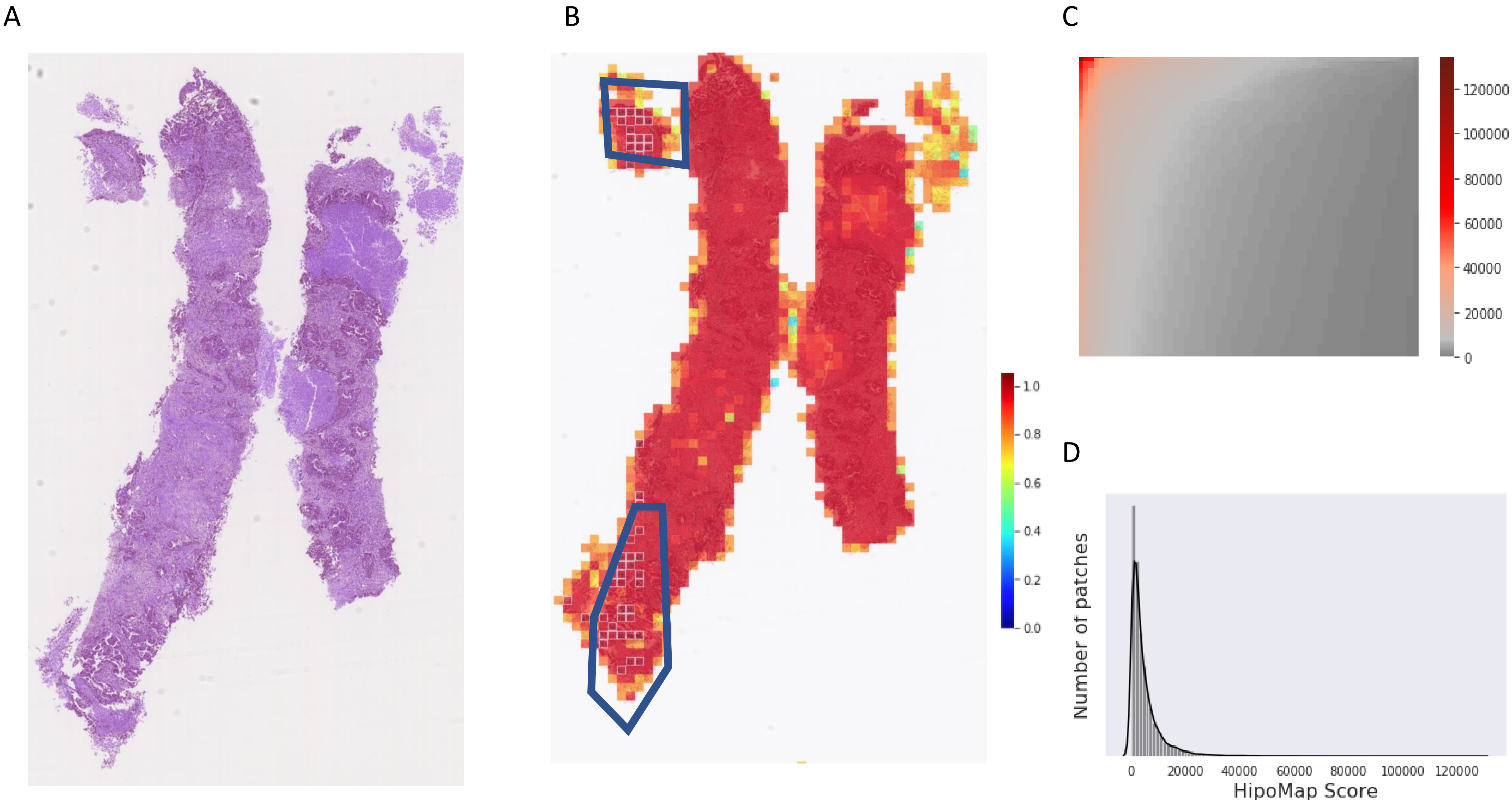

Figure S2. (A) The original WSI, (B) patch-wise probability map, (C) HipoMap, and D) histogram of HipoMap score ADC-183-1052.

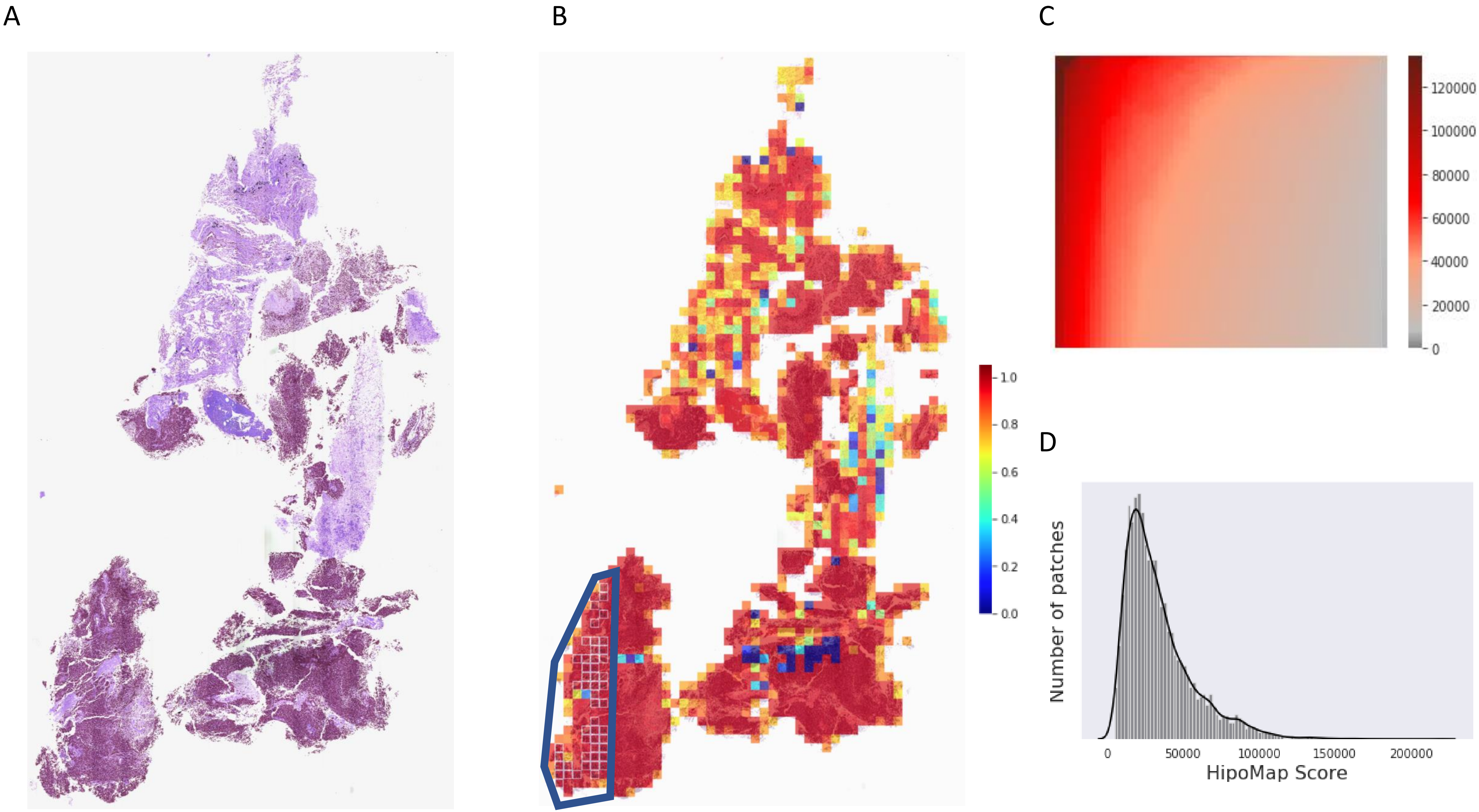

Figure S3. (A) The original WSI, (B) patch-wise probability map, (C) HipoMap, and D) histogram of HipoMap score SCLC-139-1772.

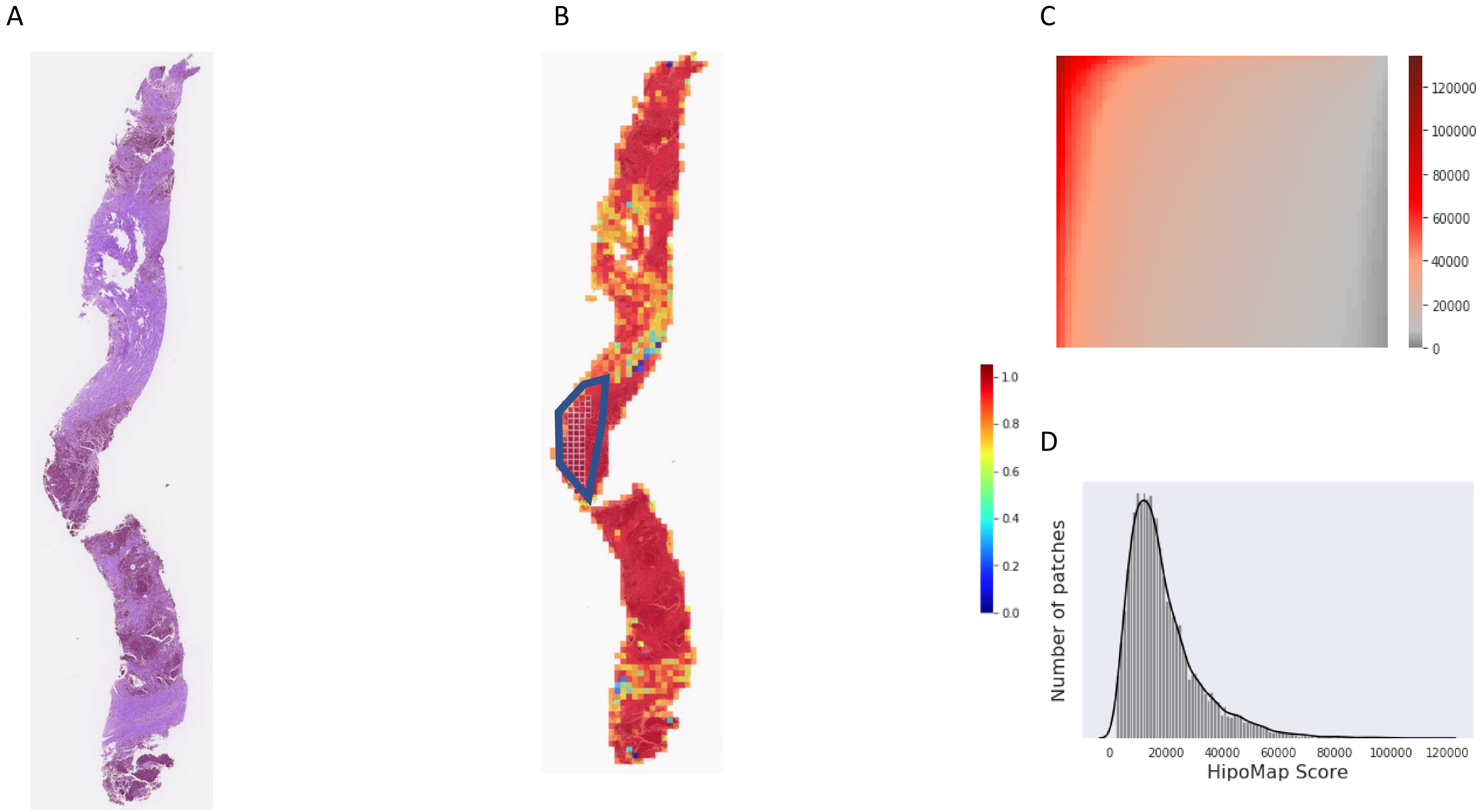

Figure S4. (A) The original WSI, (B) patch-wise probability map, (C) HipoMap, and D) histogram of HipoMap score LCNEC-194-1537.

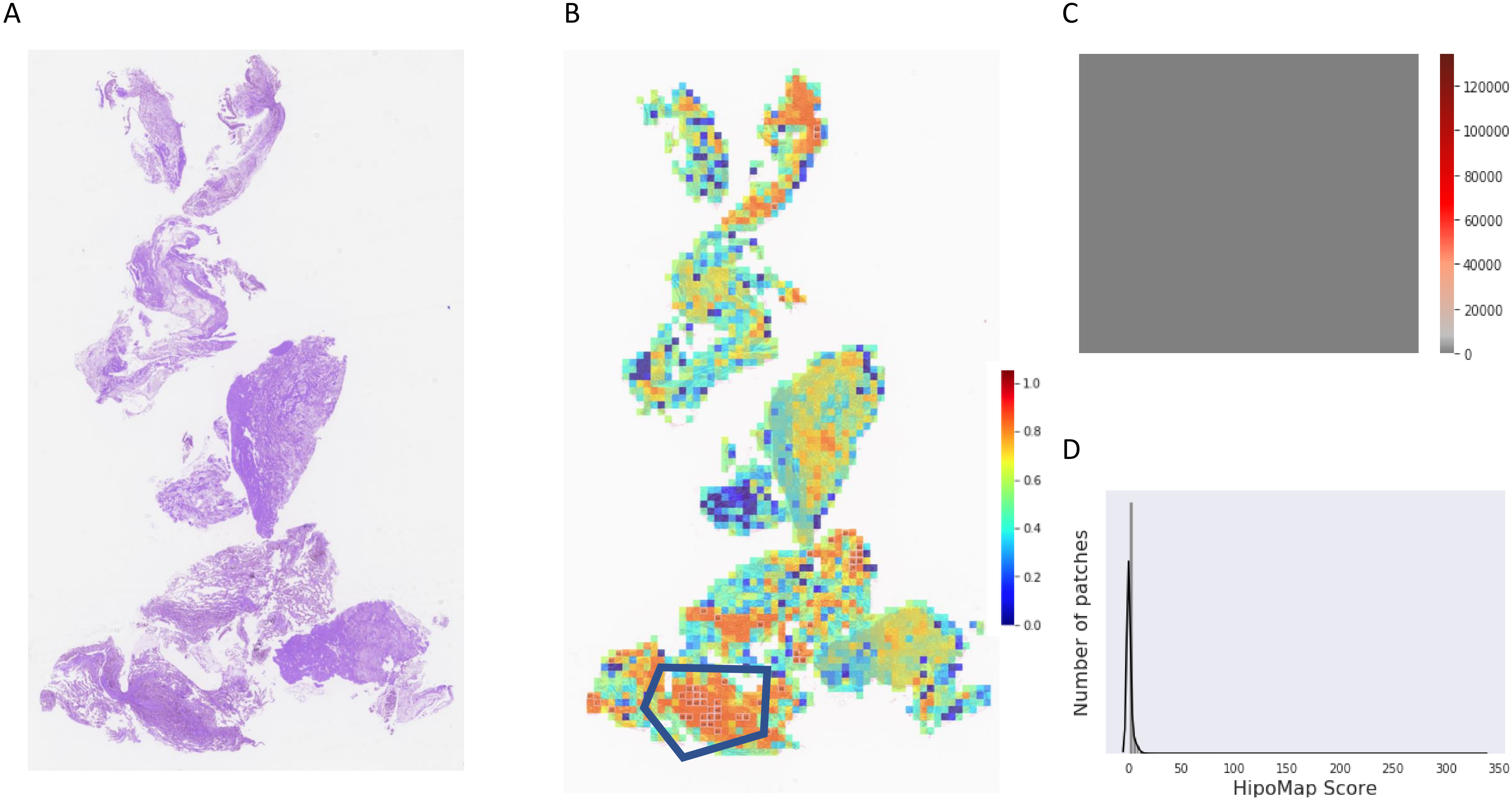

Figure S5. (A) The original WSI, (B) patch-wise probability map, (C) HipoMap, and D) histogram of HipoMap score N-155-1721.

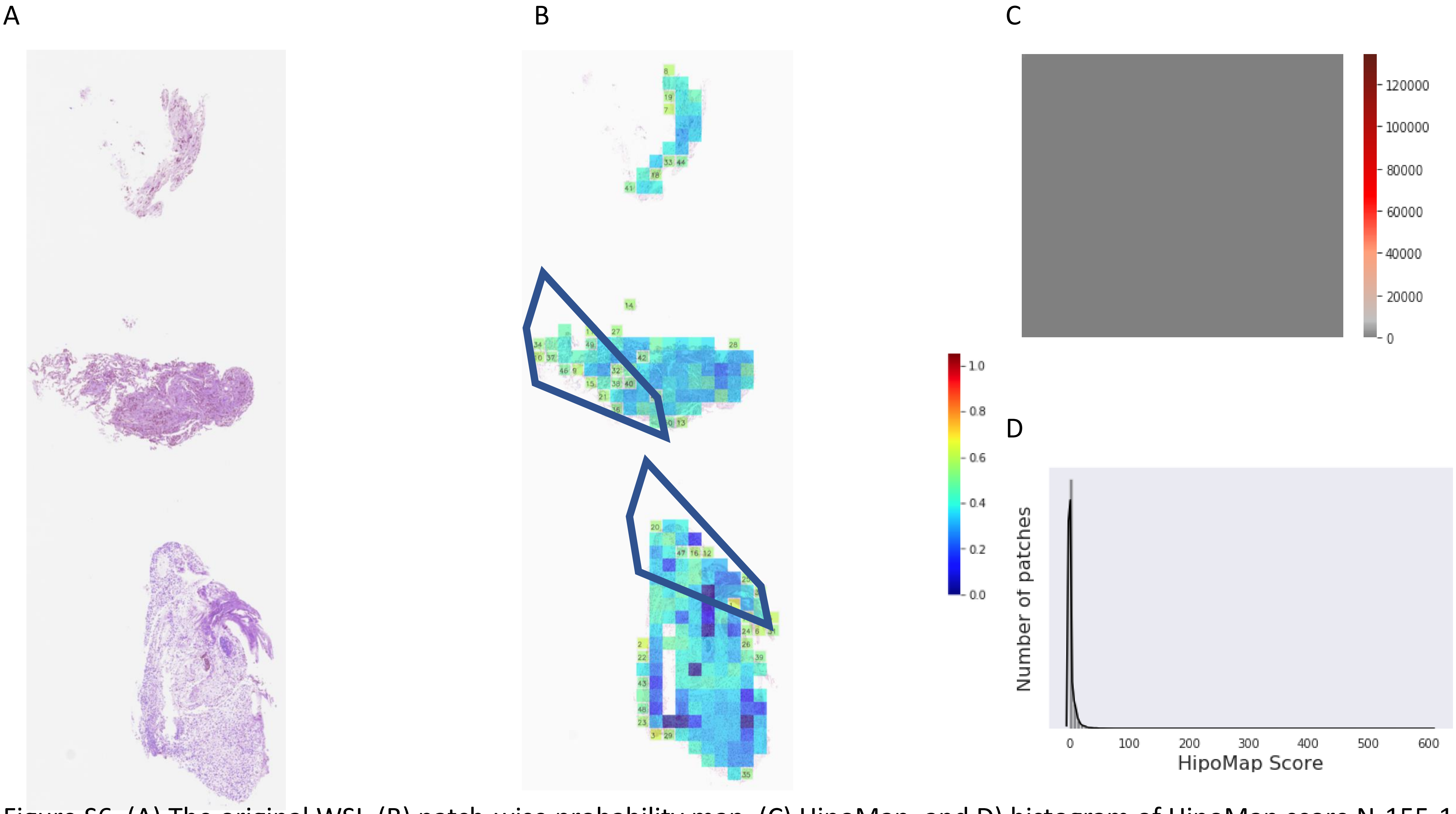

Figure S6. (A) The original WSI, (B) patch-wise probability map, (C) HipoMap, and D) histogram of HipoMap score N-155-1552.

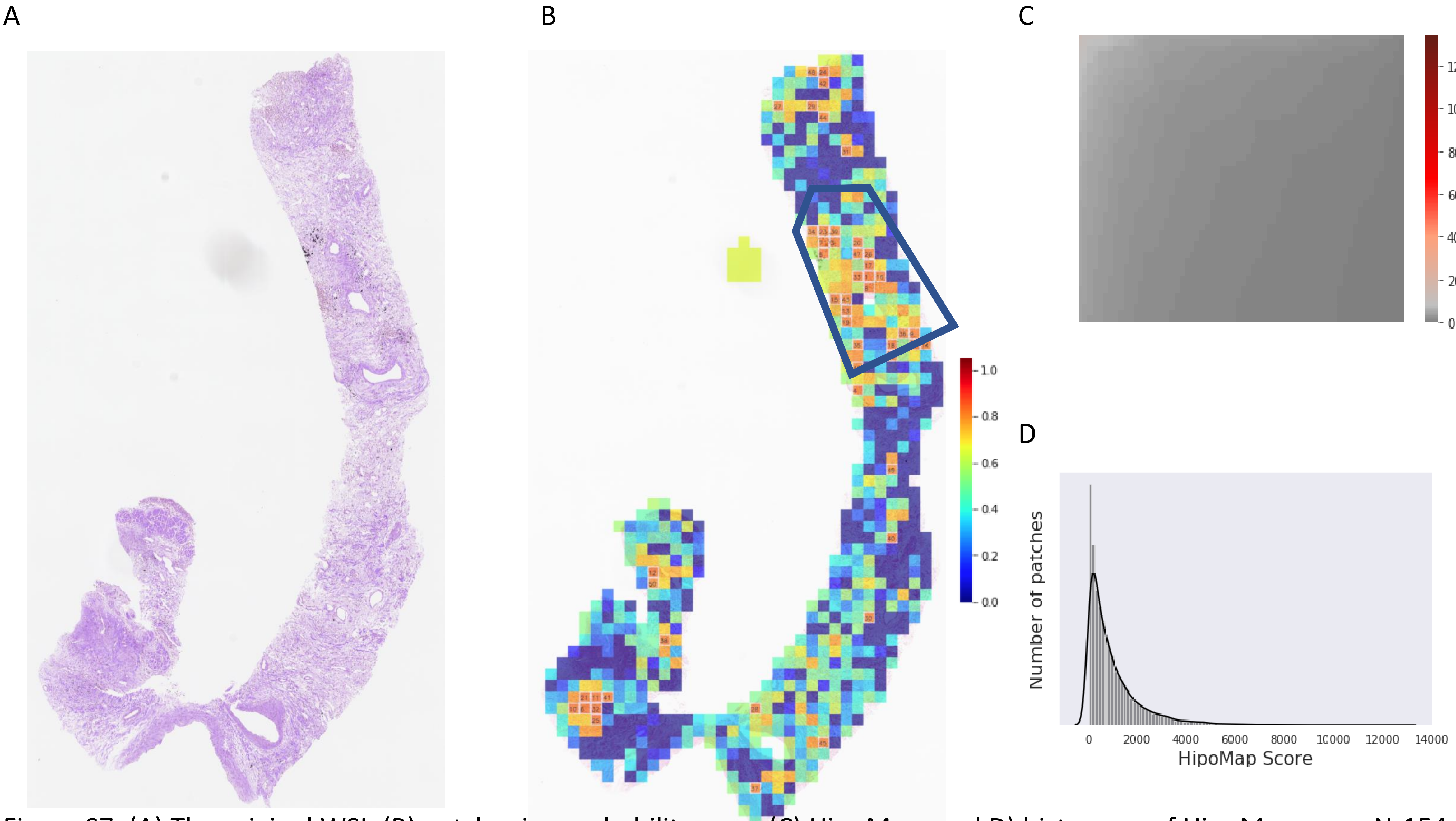

Figure S7. (A) The original WSI, (B) patch-wise probability map, (C) HipoMap, and D) histogram of HipoMap score N-154-1322.

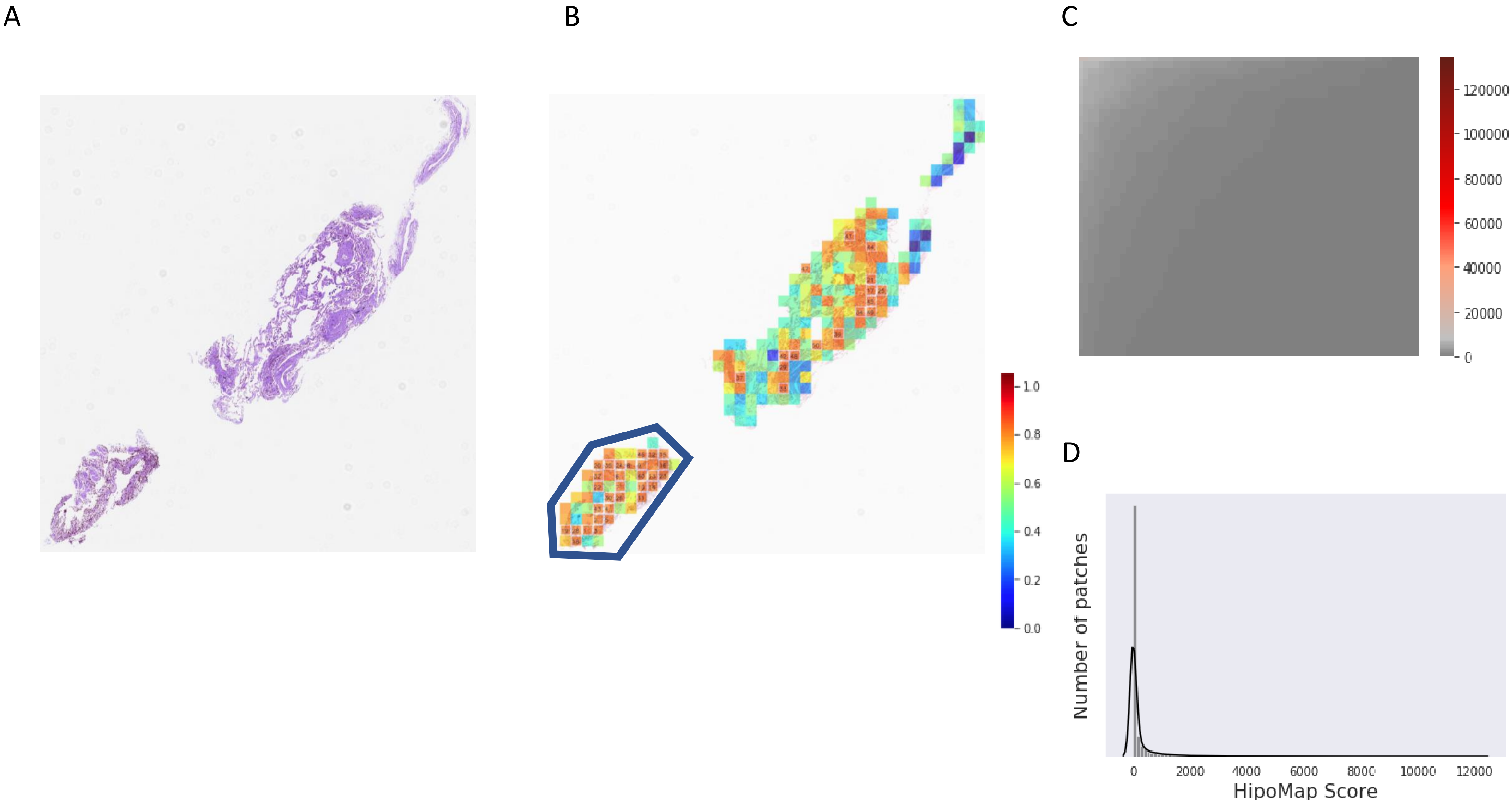

Figure S8. (A) The original WSI, (B) patch-wise probability map, (C) HipoMap, and D) histogram of HipoMap score N-155-1465.

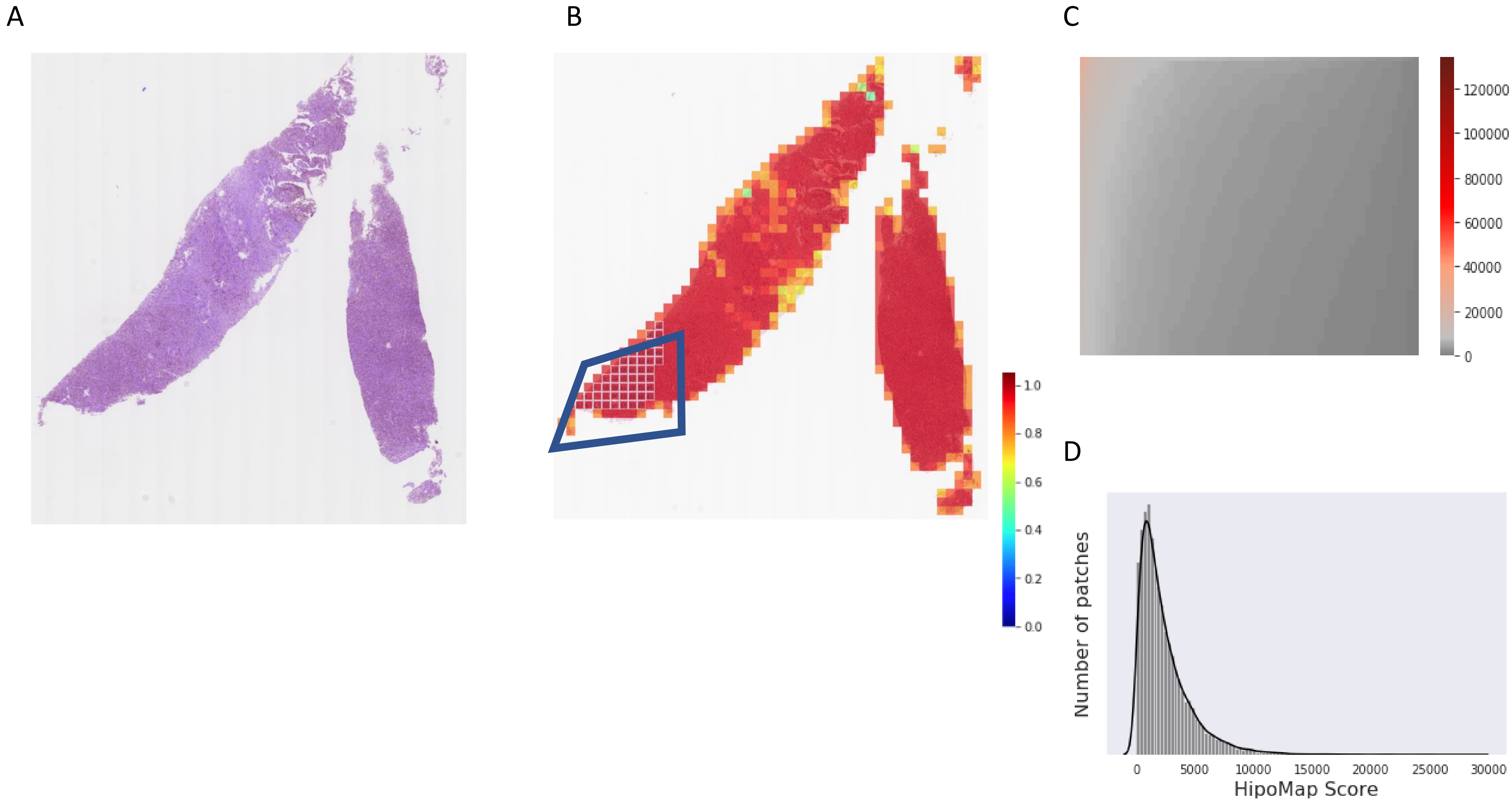

Figure S9. (A) The original WSI, (B) patch-wise probability map, (C) HipoMap, and D) histogram of HipoMap score N-681-1513.

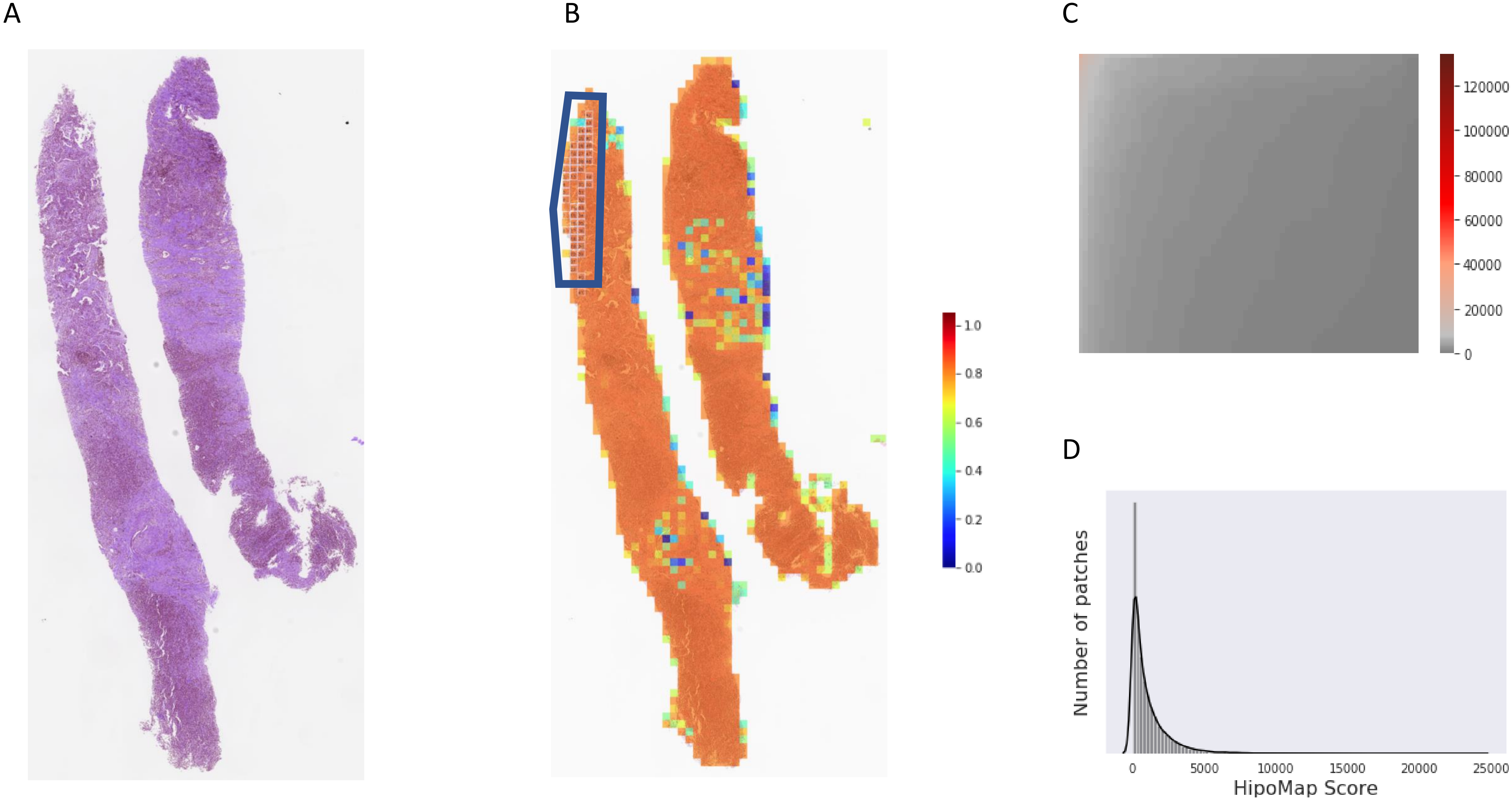

Figure S10. (A) The original WSI, (B) patch-wise probability map, (C) HipoMap, and D) histogram of HipoMap score N-237-3069.

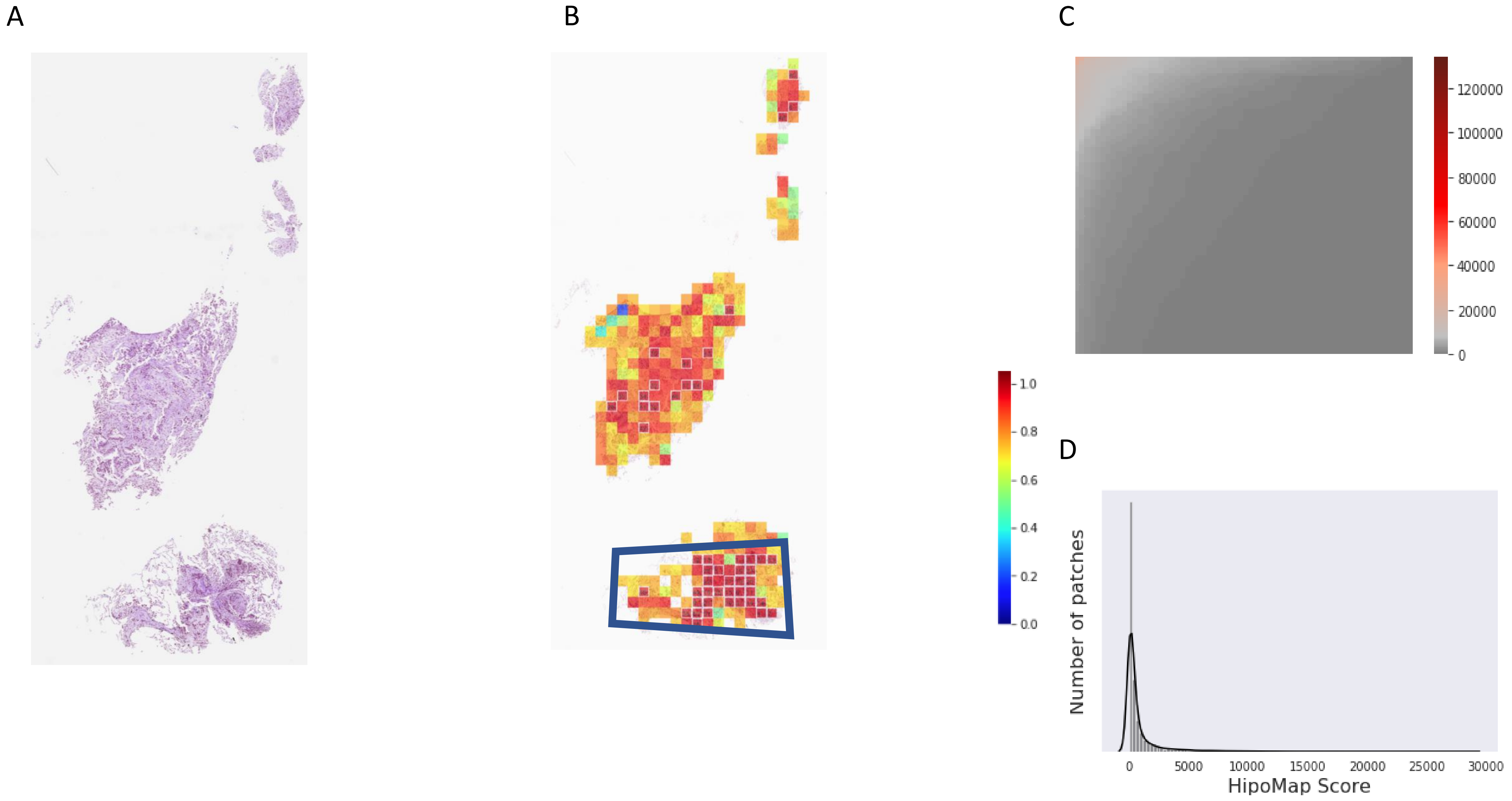

Figure S11. (A) The original WSI, (B) patch-wise probability map, (C) HipoMap, and D) histogram of HipoMap score ADC-951-8686.
